# Marine Natural Products from Flora and Fauna of the Western Australian Coast: Taxonomy, Isolation and Biological Activity

**DOI:** 10.3390/molecules28031452

**Published:** 2023-02-02

**Authors:** Samuele Sala, Scott K. Micke, Gavin R. Flematti

**Affiliations:** 1 School of Molecular Sciences, The University of Western Australia, Crawley, WA 6009, Australia; 2 Australian National Phenome Centre and Centre for Computational and Systems Medicine, Health Futures Institute, Murdoch University, Harry Perkins Building, Perth, WA 6150, Australia

**Keywords:** secondary metabolites, marine natural products, flora, fauna, Western Australia

## Abstract

Marine natural products occurring along the Western Australian coastline are the focus of this review. Western Australia covers one-third of the Australian coast, from tropical waters in the far north of the state to cooler temperate and Antarctic waters in the south. Over 40 years of research has resulted in the identification of a number of different types of secondary metabolites including terpenoids, alkaloids, polyketides, fatty acid derivatives, peptides and arsenic-containing natural products. Many of these compounds have been reported to display a variety of bioactivities. A description of the compound classes and their associated bioactivities from marine organisms found along the Western Australian coastline is presented.

## 1. Introduction

Natural products have long played an important role both as direct agents and as molecular scaffolds providing inspiration for novel pharmaceuticals [1]. Notably, over 60% of all agents used currently in the treatment of cancer can be traced back to a natural product source [1]. Similarly, nearly 50% of all anti-bacterial agents and all anti-parasitic small molecules are either natural products or natural-product-derived compounds [2], highlighting the importance of natural product discovery as a source of many different pharmaceutical agents.

Historically, marine natural product research has lagged behind its terrestrial counterpart due to the inaccessibility of samples, as well as the absence of ethnobotanical knowledge in guiding the selection of taxa for investigation [3]. The development of the field in the 1950s coincided with the mass natural product screening campaigns conducted by the National Cancer Institute (NCI) in the United States, as well as the development of SCUBA (Self-Contained Under-water Breathing Apparatus) technology, and later remotely operated vehicles that allowed natural product chemists unprecedented access to unfamiliar benthic biomes. More recently, significant advances in the field have been propelled by developments in tools for identifying small molecules such as high-resolution mass spectrometry (HR-MS) coupled to high-performance and ultra-high-performance liquid chromatography, as well as advances in high-resolution nuclear magnetic resonance (NMR) spectroscopy [3]. The past decades have also seen significant pharmaceutical interest in the discovery of novel drug entities from marine sources [2,4].

Western Australia covers one-third of Australia’s coast, from tropical waters in the far north of the state to cooler temperate and Antarctic waters in the state’s south [5]. The state has a topographically diverse continental margin, with features of the continental shelf including coarse sediments in the south of the state around Point Hillier and Bald Island with large rocky banks in the central western region around Houtman Abrolhos and a deep continental shelf in the north of the state. The central continental shelf features a number of deep submarine canyons off Perth, Two Rocks and Kalbarri [5]. The sponge gardens of Ningaloo Reef, Carnarvon shelf, have been denoted as biodiversity hotspots with an estimated 840 unique inhabitant sponge species [6].

Marine natural product research on taxa of the Western Australian coast was spear-headed in the late 1970s and early 1980s by then PhD student Robert J. Capon, under the tutelage of Prof. Emilio L. Ghisalberti and Prof. Phillip R. Jefferies at the University of Western Australia (UWA), investigating the secondary metabolite constituents of marine sponges and macro-algae of the South-Western Australian coast. In parallel to this was the work of Dr. Kevin Francesconi and Dr. John S. Edmonds at the Western Australian Marine Research Laboratories and later Prof Robert V. Stick and co-workers at UWA, investigating the sequestration and metabolism of elemental arsenic within the marine food web. Subsequent research efforts beginning in the 1990s were led by the research group of now Prof. Robert Capon, during his various affiliations with the University of Melbourne (UM) and the University of Queensland (UQ), analysing the secondary metabolomes of marine invertebrates recovered from scientific trawling expeditions conducted over the southwest of Western Australia and the Great Australian Bight. It was during this period that the research group of Prof. Tadeusz F. Molinski, at the University of California San Diego (UCSD), made significant inroads into the marine sponges of Ningaloo Reef and the Exmouth Gulf, culminating in the isolation of the phorboxazoles A and B (**150**, **151**) from the marine sponge *Phorbas* sp. [7], at the time of isolation the second most-potent cytotoxic agents tested against the National Cancer Institute’s 60-cell-line screen. Also notable during this time was the isolation of the salicylihalamides A and B (**163**, **164**) from a *Haliclona* sp. [8] by Michael R. Boyd and co-workers affiliated with the NCI, significant for their unprecedented mechanism of action via Vacuolar-ATPase inhibition [9]. Finally, prominent investigations during this period were also conducted in the laboratory of Prof. William Fenical at Scripps Institute of Oceanography, UCSD, investigating the secondary metabolites of tunicates and soft corals of the Indian Ocean and Western Australian Coast, notably leading to the isolation of eleutherobin (**223**), isolated from the Alcyonacaen soft coral *Eleutherobia* sp. [10].

The following review attempts to provide a comprehensive account of all natural products isolated from Western Australian waters as of December 2022, as well as an account of associated bioactivities and relevant taxonomic information. Articles relevant to the review were found using the MarinLit [11] database’s geographical search function and pursuit of any subsequent literature. Sections have been divided taxonomically. In the case of Porifera, necessitated by the extensive number of reported compounds, sub-sections have been further divided by presumed biosynthetic class: in the case of evident mixed biogenesis, compounds have been arbitrarily assigned to a relevant sub-section. Within subsections, an attempt has been made to detail the isolation of respective natural products chronologically. Moreover, for the sake of coherence, arsenic metabolites have been devoted their own section at the end of this review. Any work omitted from this review was unintentional on the part of the authors.

## 2. Discussion

### 2.1. Porifera

#### 2.1.1. Terpenoids

**Figure 1:**
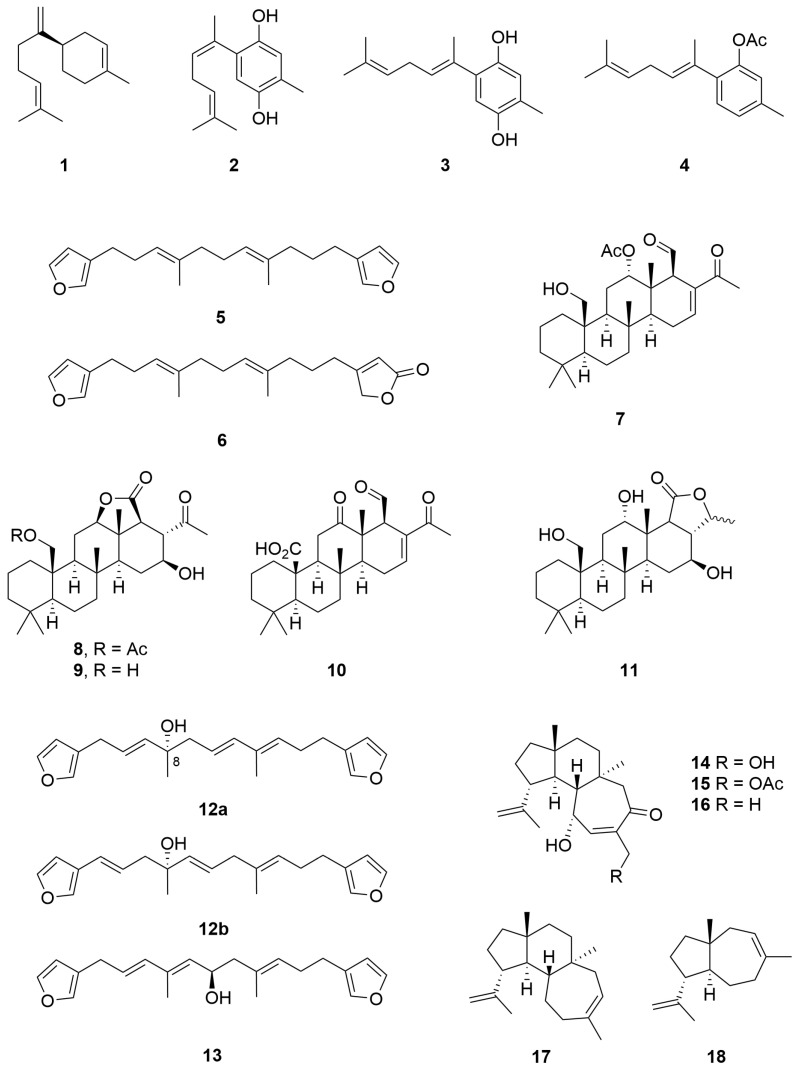
Compounds **1** to **18**.

Three aromatic sesquiterpenes, as well as the known compound (−)-bisabolene (**1**), were isolated from the non-polar fractions of a *Halichondria* sp. (Order: Suberitida; Family: Halichondridae) collected off the coast of Lancellin. The unknown compounds were identified as (1′*Z*)- and (1′*E*)- 2-(1′,5′-dimethylhexa-4′-dienyl)-5-methylbenzene-1,4-diol (**2**, **3**) and (1′*E*)-2-(1′,5′-dimethylhexa-1′,4′-dienyl)-5-methyl-phenyl acetate (**4**) (Figure 1) using ^1^H NMR and ^13^C NMR spectroscopy as well as chemical interconversion [12].

A *Lendenfeldia* sp. (Order: Dictyoceratida; Family: Thorectidae) specimen collected at Quobba Lagoon was the source of two known C-21 furanoterpenes (**5**, **6**) as well as five new C-26 scalarene sesterterpenes (**7**–**11**) (Figure 1). The compounds were characterised spectroscopically as well as via chemical derivatisation and comparison to earlier reports. Two of the previously reported scalarenes and the novel compound **8** exhibited extremely potent inhibition of platelet aggregation, this providing a rationalisation for the anti-inflammatory activity of this group of compounds [13]. 

A *Spongia* sp. (Order: Dictyoceratida; Family: Spongiidae) collected east of Gun Island, South Abrolohos Group, was the source of a new C-21 bisfuranoterpene bearing a tertiary hydroxyl at position C-8 as an unstable oil (**12a**) [14]. The structure of the natural product was subsequently revised to **12b** following two-dimensional NMR analysis [15]. The former publication also reports the revised stereochemistry via the Horeau method of another bisfuranoterpene **13** isolated from a *Leiosella* sp. collected by dredge off Rottnest Island (Figure 1) [14]. 

Three tricyclic diterpenes **14**–**16** were reported from a collection of *Higginsia* sp. (Order: Axinellida; Family: Stelligeridae) collected off Lancelin. The structure of **14** was verified via single-crystal X-ray diffraction [16]. The structures of the related monoacetate and monoalcohol were deduced in relation to that of **16**. Subsequent reinvestigation of the non-polar fractions of the lipophilic sponge extract afforded the tricyclic diterpene hydrocarbon **17** and the daucadiene sesquiterpene **18** (Figure 1) [17]. A biosynthetic scheme arising from farnesyl and geranylgeranyl pyrophosphate was proposed. Furthermore, the authors suggest that compounds **14** to **16** derive via oxidation of **17**, followed by intermolecular 4 + 2 cycloaddition of oxygen.

**Figure 2:**
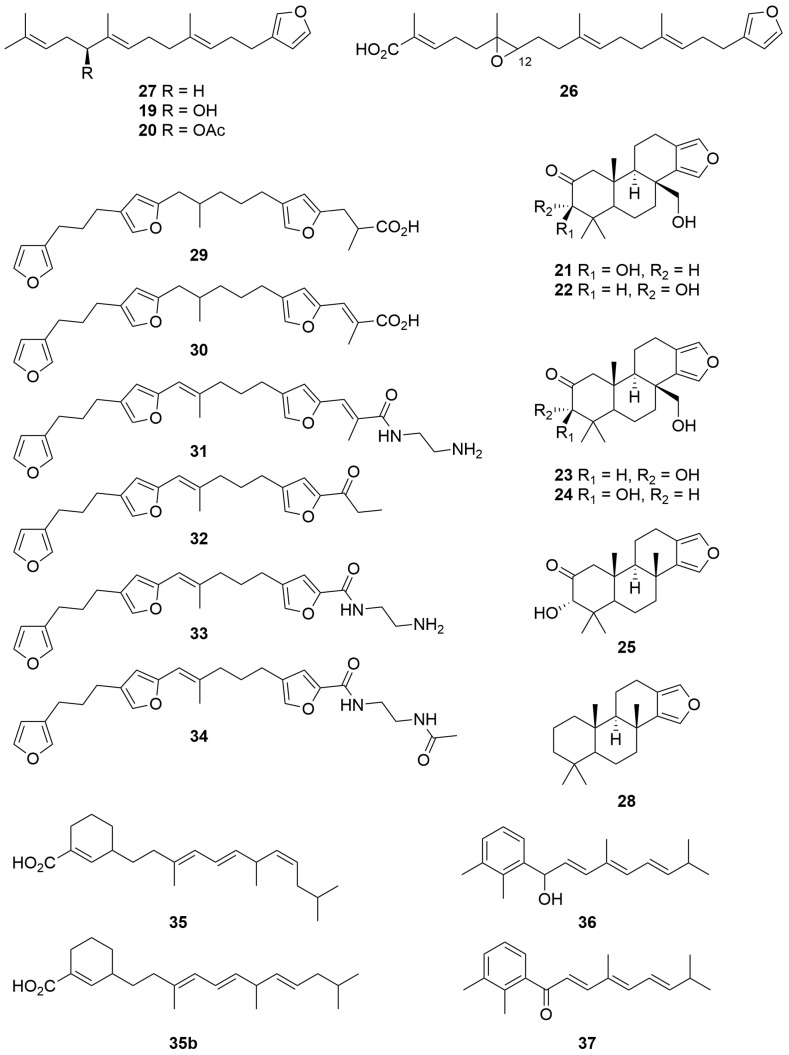
Compounds **19** to **37**.

A *Spongia* sp. (Order: Dictyoceratida; Family: Spongiidae) collected from Exmouth gulf afforded the novel linear furanoditerpenes 12-hydroxy ambliofuran (**19**) and 12-acetoxyambliofuran (**20**) (Figure 2) [18]. Mosher’s ester analysis revealed the compounds to be a scalemic mixture of 3:1, predominantly *S* configured enantiomers: the authors note that the isolation of enantiomers in non-racemic proportions is unusual in the field of marine natural products. Additional investigations of the sponge extract unearthed the new tetracyclic furanoditerpenes **21**–**25** and the linear furanosesterpene **26** bearing an epoxide at C-12, as well as the known compounds **27** and **28** (Figure 2) [18].

The CH_2_Cl_2_ soluble fractions of a *Hippospongia* sp. (Order: Dictyoceratida; Family: Spongiidae) collected from south of the Great Australian Bight afforded six new C-25 derived linear furanoterpenes, given the trivial names hippospongins A–F (**29**–**34**) (Figure 2) [19]. The authors postulate a biosynthetic link between the commonly encountered C-25 tetronic acids and C-21 furanoditerpenes commonly encountered in marine sponges via the intermediacy of compounds **29**–**34**. Hippospongin A (**29**) exhibited mild antibiotic activity, inhibiting the growth of *Staphylococcus aureus* at concentrations of *circa* 200 µg/disk in a standard agar plate assay [19].

An investigation into the chemistry of a *Clathria* sp. (Order: Poecilosclerida; Family: Microcionidae) collected off the Great Australian Bight yielded the novel compounds clathrins A, B and C (**35**–**37**) (Figure 2). Clathrin A (**35**) is postulated to provide support for the biosynthetic origins of other marine meroterpenoids derived via a mixed shikimate-terpenoid biosynthetic pathway. Attempts to elucidate the stereochemistry of clathrin B (**36**) were thwarted by the facile atmospheric oxidation of **36** to compound **37**; furthermore, the absolute configuration of **35** remains unresolved [20].

**Figure 3:**
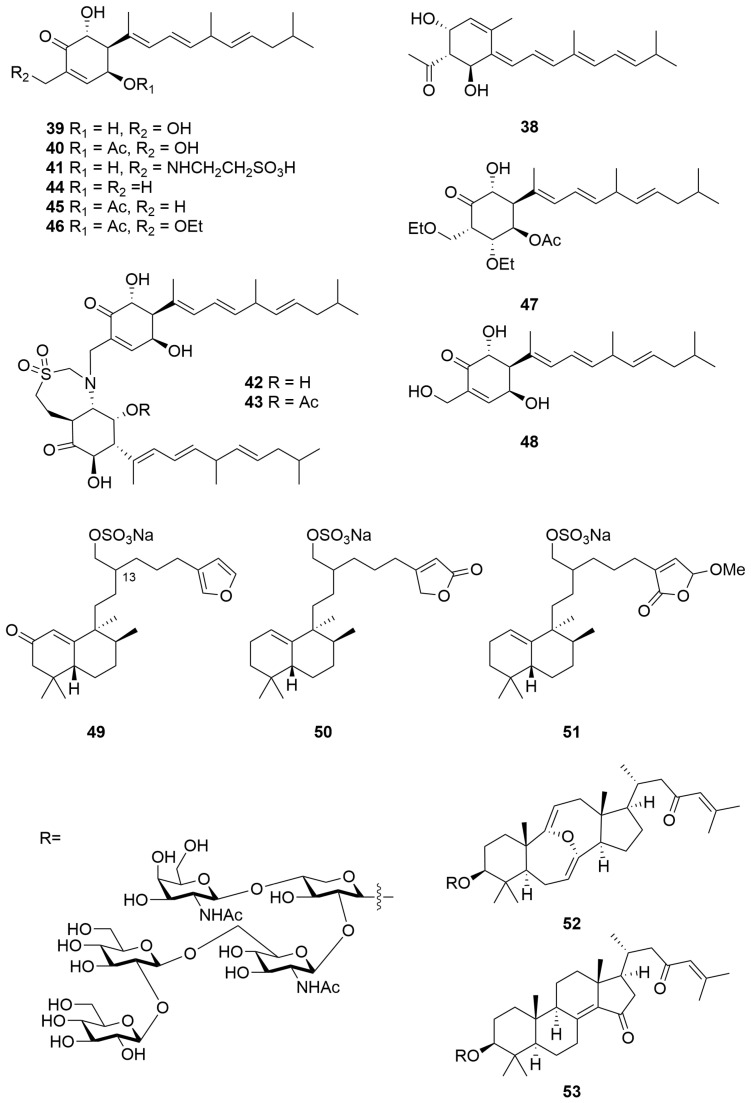
Compounds **38** to **53**.

The ethanolic extracts of a *Phorbas* sp. (Order: Poecilosclerida; Family: Hymedismiidae) sourced from the Great Australian Bight afforded the rearranged diterpenoid phorbasin A (**38**) (Figure 3) as an unstable pale yellow solid; the unprecedented carbon skeleton of **38** was elucidated spectroscopically [21]. Analysis of a second *Phorbas* sp. unearthed phorbasins B and C [22], which were subsequently revised to structures **39** and **40** (Figure 3) with a terminal *E*, instead of *Z*, configured double bond [23]. (In light of the reassignment of phorbasins A and B to **39** and **40**, we postulate that clathrin A (**35**) [20] may also require revision to structure **35b** with all *E* configured olefins.) Further analysis of the sponge genus unearthed phorbasins D–F (**41**–**43**) (Figure 3), the dimeric compounds **42** and **43** bearing an unusual taurine-conjugated seven-membered heterocycle [23]. Research efforts into the phorbasin class culminated in the isolation of phorbasins G–K (**44**–**48**) [24]. The authors postulate a likely artefactual origin for phorbasins I (**46**) and J (**47**) via solvolysis [24]. The crude EtOH extract exhibited cytotoxic activity, as well as growth-inhibitory activity against the Gram-positive bacteria *Staphylococcus aureus* and *Micrococcus luteus*. Phorbasins B (**39**) and C (**40**) (Figure 3) were determined to be the principal antibacterial constituents. Compounds **39**, **40**, **44**, **45** and **46** exhibited substantial potency and selective cytotoxicity against Neonatal Foreskin Fibroblasts (NFF) and human cancer (A549, HT29 and MM96L) cell lines. The structure–activity relationship of the metabolites was found to correlate with the presence of an α,β-unsaturated ketone functionality. 

Chemical investigations of a *Darwinella australensis* (Order: Dendroceratida; Family: Darwinellidae) collected by SCUBA in the East Timor Sea afforded three new sesterpene sulfates, halisulfates 8–10 (**49**–**51**) (Figure 3) [25], isolated as their sodium salts. The relative configurations of the decalin moiety were elucidated using combined spectroscopic methods and via comparison to the known halisulfates 1–7. The relative configuration at C-13 remains unresolved. Halisulfates 9 (**50**) and 10 (**51**) exhibited inhibition of cell division of sea urchin eggs (*Strongylocentrotus intermedius*) in moderate concentration (IC_50_ = 50 µg/mL and 35 µg/mL, respectively) [25]. 

Sarasinosides A_4_ (**52**) and A_5_ (**53**) (Figure 3) were isolated from a marine sponge *Melophlus sarasinorum* (Order: Tetractinellida; Family: Geodiidae) collected near Scott Reef, along with five known sarasinosides [26]. The compounds were elucidated on the basis of extensive nuclear magnetic resonance experiments and density functional theory calculations, as well as MALDI-TOF-MS and GC-MS analysis. The compounds isolated bear the same oligosaccharide moiety and differ only in the composition of the aglycone. Compound **52** is unusual in the composition of its bis-enol ether moiety [26]. 

**Figure 4:**
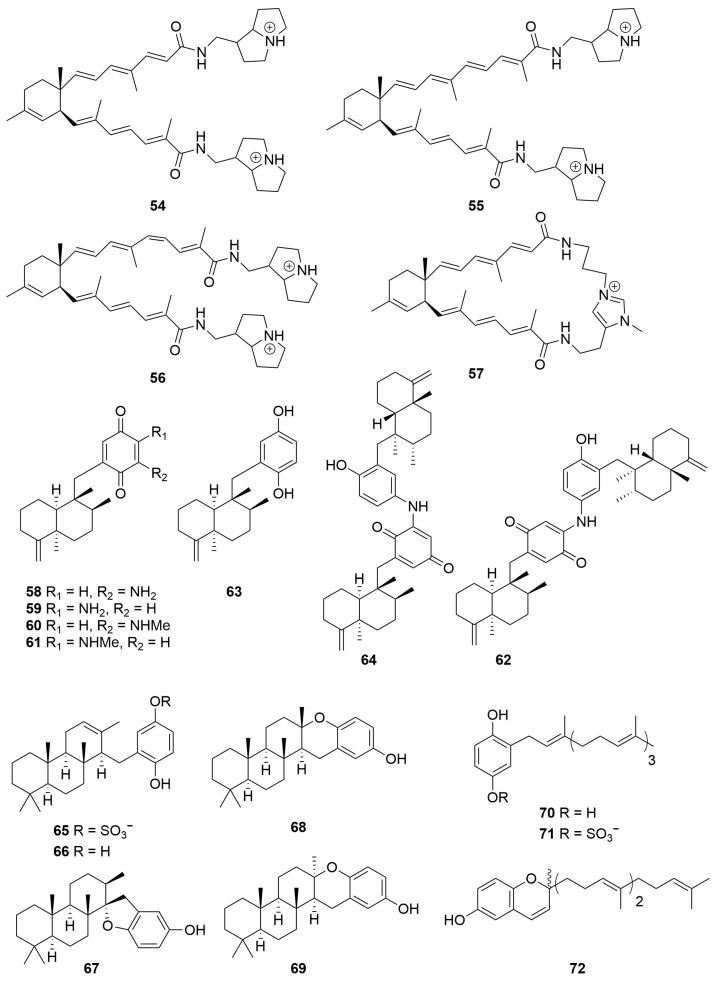
Compounds **54** to **72**.

A sample of *Stelletta* sp. (Order: Tetractinellida; Family: Ancorinidae) collected by trawling operations in the Great Australian Bight was the source of the terpenyl-pyrrolizidine conjugates bistelletazines A–C (**54**–**56**) and the cyclic terpenyl-imidazole conjugate macrocycle bistelletazole A (**57**) (Figure 4) [27]. The authors note that despite extensive two-dimensional nuclear magnetic resonance experiments performed, the data acquired did not allow for the unambiguous assignment of stereochemistry for the pyrrolizidine portion of the molecule. The authors propose the compounds to share a convergent biosynthesis, the unique carbon scaffold arising from a presumed Diels–Alder reaction between two polyene sesquiterpene precursors [27]. 

Four meroterpenoid pigments, 18-aminoarenarone (**58**), 19-aminoarenarone (**59**), 18-methylaminoarenarone (**60**) and 19-methylaminoarenarone (**61**), and the new dimeric popolohuanone F (**62**) were isolated from a *Dysidea* sp. (Order: Dictyoceratida; Family: Dysideidae) collected from Scott Reef [28]. The sample also afforded the known compounds arenarol (**63**) and popolohuanone A (**64**) (Figure 4). Further investigation revealed that **62**, **63** and **64** exhibited DPPH radical scavenging activity with IC_50_ values of 35 μM, 35 μM and 19 μM, respectively [28].

A new meroterpeneoid sulfate, fascioquinol A (**65**), was isolated from a deep-water *Fasciospongia* sp. (Order: Dictyoceratida; Family: Thorectidae) along with its desulfated counterpart fascioquinol B (**66**), and the acid-mediated cyclisation products fascioquinols C, D and strongylophorine-22 (**67**–**69**). The sponge also afforded the known meroterpene geranylgeranyl-1,4-hydroquinone (**70**) and its sulfated counterpart, to which was assigned the trivial name fascioquinol E (**71**), as well as the racemic chromenol fascioquinol F (**72**) (Figure 4) [29]. Further investigation revealed that **70** exhibited specific cytotoxic activity against gastric adenocarcinoma (AGS, IC_50_ = 8 µM) and neuroblastoma (SH-SY5Y, IC_50_ = 4 µM) cell lines; in addition to this, **65** and **66** exhibited promising Gram-positive activity towards *Staphylococcus aureus* (IC_50_ = 0.9–2.5 µM) and *Bacillus subtilis* (IC_50_ = 0.3–7.0 µM).

#### 2.1.2. Alkaloids

**Figure 5:**
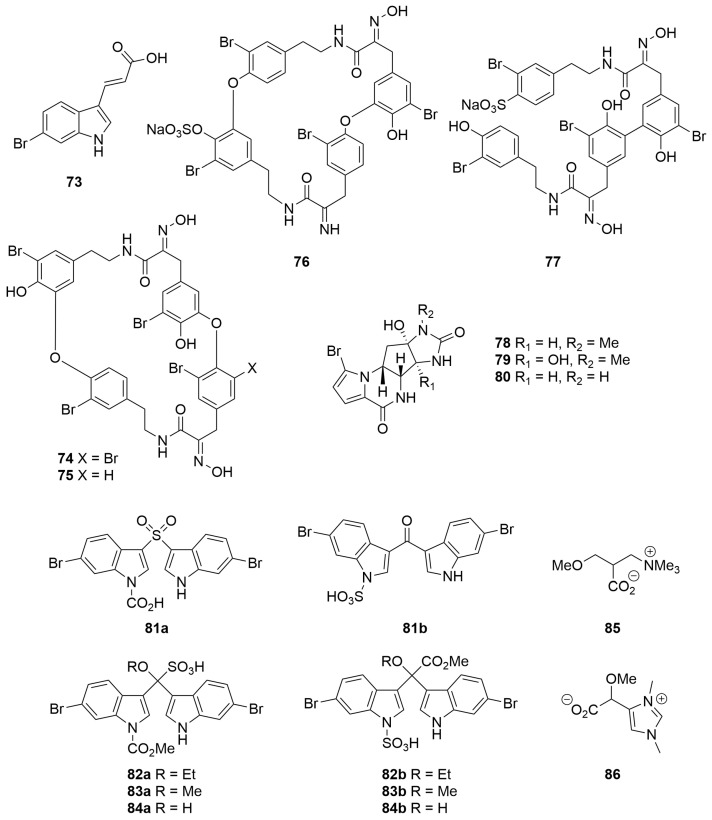
Compounds **73** to **86**.

An *Iotrochota* sp. (Order: Poecilosclerida; Family: Iotrochotidae) collected from the Five Fathom Bank, off the coast of Fremantle, was reported to yield the novel metabolite (*E*)-3-(6-bromoindol-3-yl)prop-2-enoate (**73**) (Figure 5) [30]. The structure of the metabolite was proposed based on MS, IR and ^1^H NMR analysis and confirmed via total synthesis from 4-bromo-2-nitro-toluene [30].

Four novel bromo-tyrosine alkaloids of the bastadin class, bastadin 19 (9-debromobastadin 13, **74**), bastadin 20 (**75**) and the sulfate half-esters 15,34-O-bis-sulfatobastadin 7 (**76**) and 10-*O*-sulfatobastadin 3 (**77**) (Figure 5), were isolated from the polar fractions of a *Ianthella basta* (Order: Verongiida; Family: Ianthellidae) collected from Stuarts Shoal, Exmouth Gulf [31]. The sponge also afforded a number of other known bastadins. The authors propose the use of MALDI-MS and a microscale derivatisation combined with ^1^H NMR fingerprinting of permethylated derivatives in order to rapidly dereplicate known bastadin and isobastarane isomers [31]. The compounds 15,34-*O*-disulfatobastadin 7 (**76**) and 10-*O*-sulfatobastadin 3 (**77**) exhibited moderate and specific activity as Sarcoplasmic Reticulum (SR) Ca^2+^ channel agonists (EC_50_ = 13.6 µM and 100 µM, respectively) of the Ry1R FKBP12 complex [31].

The methanolic extracts of two samples of *Cymbastela* sp. (Order: Axinellida; Family: Axinellidae) collected by SCUBA near Muiron Island afforded the known compound agelastin A (**78**) and the novel analogues agelastins C and D (**79**, **80**) (Figure 5) [32]. The structures of the compounds were determined spectroscopically and via chemical derivatisation. Agelastatin A (**78**) exhibited potent activity against brine shrimp (LC_50_ = 5.0 µM) in addition to potent insecticidal activity against larvae of beet army worm, *Spodoptera exigua*, and corn rootworm, *Diabrotica undecimpunctata* [32].

The ethanolic extracts of an *Echinodictyum* sp. (Order: Axinellida; Family: Raspailiidae) collected in the Great Australian Bight afforded four novel compounds, echinosulfone A (**81a**) and the echinosulfonic acids A–C (**82a**–**84a**) (Figure 5) [33]. The proposed structures were assigned based on extensive two-dimensional NMR analysis. The compounds were found to account for the antibacterial activity of the crude extract but not the reported nematocidal activity. The structures of echinosulfone A and the echinosulfonic acids A–C were subsequently revised by three independent research groups contemporaneously to structures **81b**–**84b** (Figure 5), respectively, on the basis of synthetic efforts, as well as single-crystal X-ray diffraction and density functional theory analysis [34,35,36]. Subsequent bioassay-guided fractionation of the sponge extract unearthed the novel betaine alkaloids (−)-echinobetaine A (**85**) [37] and (+)-echinobetaine B (**86**) (Figure 5) [38] as the principal nematocidal components responsible for the bioactivity of the sponge crude extract against the commercial livestock parasite *Haemonchus contortus* with echinobetaine B (**86**) exhibiting an LD_99_ of 8.3 µg/mL. The structures of racemic echinobetaines A and B have also been confirmed via total synthesis [38].

**Figure 6:**
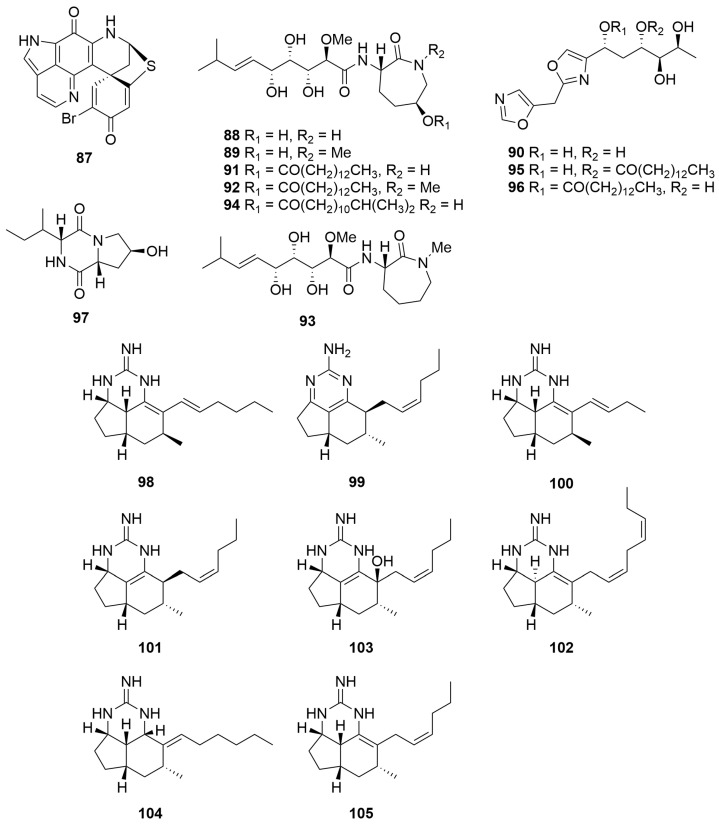
Compounds **87** to **105**.

A *Zyzzya* sp. (Order: Poecilosclerida; Family: Acarnidae) collected at Assail Bank, between North Island and the Wallab Group, and a *Latrunculia purpurea* (Order: Poecilosclerida; Family: Latrunculiidae) collected on Horse Shoe Reef, west-northwest of Margaret Brock Lighthouse, were both reported to yield the novel pyrolloiminoquinone pigment discorhabdin Q (**87**) (Figure 6) [39]. The authors note that compound **87** was found not to be the principle cytotoxin in any of the extracts assayed; however, the metabolite exhibited moderate generalised cytotoxicity (mean panel GI_50_ = 0.5 µg/mL) in the NCI 60 cancer cell line panel [39].

A *Jaspis* sp. (Order: Tetractinellida; Family: Ancorinidae) collected off a low, uninhabited rocky island near the northwestern end of Serrurier Island, afforded the novel alkaloids bengamides Y (**88**) and Z (**89**) and bengazole Z (**90**) (Figure 6) [40]. In the same report, the authors detail that reinvestigation of the non-polar fractions yielded the known metabolites bengamides A (**91**) and B (**92**). Biological investigation revealed that metabolites **88** and **89** exhibited specific cytotoxicity against 10 cancer cell lines [40]. Bioassay-guided fractionation of a *Stelleta* sp. (Order: Tetractinellida; Family: Ancorinidae) collected off the western side of Jamieson Reef, Bonaparte Archipelago, afforded the known metabolites bengamides A (**91**), F (**93**), N (**94**) and Y (**88**) and bengazoles Z (**90**), C_4_ (**95**) and C_6_ (**96**) in addition to a novel diketopiperazine, cyclo-(4-*S*-hydroxy-*R*-proline-*R*-isoleucine) (**97**) [41]. The relative configuration of **97** was determined spectroscopically with the aid of molecular modelling software. Cyclo-(4-*S*-hydroxy-*R*-proline-*R*-isoleucine) (**97**) exhibited minimal activity towards MCF-7, H460 and HT-29 cells (GI_50_ > 200 µM) and no activity towards SF-268 or CHO-K1 cells. In contrast, the GI_50_ values for **88**, **90**, **91**, **93**, **94**, **95** and **96** were comparable to those reported in previous studies [41].

A *Clathria* sp. (Order: Poecilosclerida; Family: Microcionidae) collected by trawl off the coast of Cape Arid yielded the new tricyclic guanidine alkaloid mirabilin G (**98**) (Figure 6) [42]. Reinvestigation of the same specimen afforded the known mirabilins C (**99**) and F (**100**) and the novel mirabilins H, I and J (**101**–**103**) [43]. Mirabilins C and F (**99**, **100**) were characterised for the first time as underivatised natural products. The absolute stereochemistry of mirabilin F (**100**) was assigned for the first time (Figure 6). The authors propose a plausible biosynthetic route to the mirabilin, ptilocaulin and netamine alkaloids starting from polyketide precursors [43]. An *Acanthella cavernosa* (Order: Bubarida; Family: Dictyonellidae) collected in the southwest of the state was the source of the novel compound mirabilin K (**104**), the specimen also affording mirabilin G (**98**) and the related netamine M (**105**) (Figure 6) [44]. Mirabilin G (**98**) exhibited modest growth-inhibitory activity against the Gram-negative bacteria *Escherichia coli* and *Serratia marcescens* and the fungus *Saccharomyces cerevisiae*. Further investigations revealed that **98** and **105** inhibited cellular degradation of PDCD4 with EC_50_ values of 1.8 μg/mL and 2.8 μg/mL, respectively. It is noteworthy that **98** and **105** were the first reported marine natural products to stabilise PDCD4 under tumour-promoting conditions. Additional investigations revealed that **98**–**103** exhibited modest cytotoxic activity with LD_50_ values greater than 30 µM against neuroblastoma (SH-SY5Y), gastric (AGS), colorectal (HT29) and intestinal (Intestine-407) cancer cell lines.

**Figure 7:**
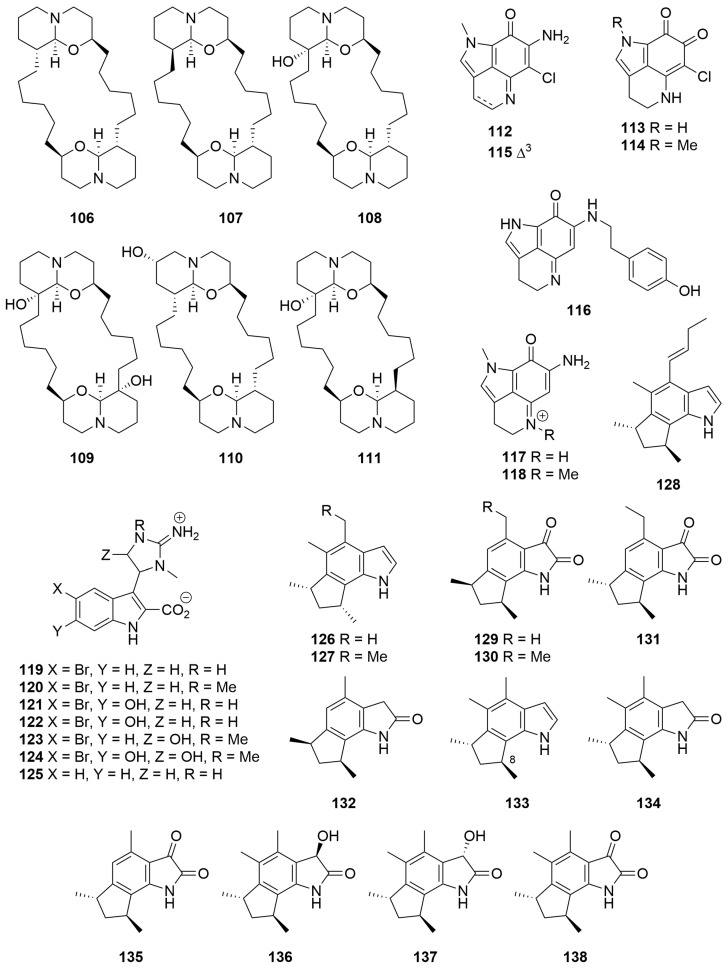
Compounds **106** to **138**.

The methanolic extracts of a *Xestospongia* sp. (Order: Haplosclerida; Family: Petrosiidae) collected at Benetts Shoal, Exmouth Gulf, afforded the dimeric 2,9-disubstituted-1-oxaquinolizidine alkaloids (+)-xestospongin A (**106**), (−)-xestospongin C (**107**) and (+)-xestospongin D (**108**), as well as arugospongine C (**109**), (+)-7*S*-hydroxyxestospongin A (**110**) and (+)-demethylxestospongin B (**111**) (Figure 7) [45]. The structure of (+)-7*S*-hydroxyxestospongin A (**110**) was solved using single-crystal X-ray diffraction, and the absolute configuration was secured using Mosher’s ester analysis. The absolute configuration of (+)-xestospongin D (**108**) was secured by analysis of anomalous dispersion in single-crystal X-ray diffraction experiments [45]. Compounds **106**–**109** and **111** exhibited modest antifungal activity (MIC 30–100 μg/mL) against various fluconazole-resistant *Candida* sp. [45]. 

Two novel pyrolloiminoquinone alkaloid pigments, isobatzelline E (**112**) and batzelline D (**113**), along with the known pigments batzelline C (**114**), isobatzelline C (**115**) and makaluvamine D (**116**) (Figure 7), were isolated from a *Zyzzya fuliginosa* (Order: Poecilosclerida; Family: Acarnidae) collected off Abrolohos Island [46]. Isobatzelline C (**115**) and to a lesser extent the known compounds makaluvamines A (**117**) and H (**118**) appear to inhibit HIV-1 envelope mediated cell fusion at concentrations less than 1.0 µg/mL [46]. 

Seven novel zwitterionic indole-2-carboxylic acids, trachycladindoles A–G (**119**–**125**) (Figure 7), were isolated from a Great Australian Bight sponge *Trachycladus laevispirulifer* (Order: Trachycladida; Family: Trachycladidae). Structures were elucidated based on comprehensive spectroscopic analysis. However, due to the paucity of material obtained, the relative configurations of trachycladindoles E (**123**) and F (**124**) remain unresolved; furthermore, the absolute configurations of **119**–**125** also remain unknown [47]. The authors postulate a biosynthetic scheme for the isolated trachycladindoles and related discodermindole family of alkaloids. Compounds **119**–**124** exhibited specific cytotoxicity against lung (A549), colorectal (HT29) and breast (MDAMB-231) cancer cell lines with GI_50_ and TGI values revealing sub µM potency. In addition to this, preliminary structure–activity relationship studies performed on compounds **119**–**125** highlighted an unusual bioactive molecular motif in favour of N-10 and N-12 dimethylation, as evidenced by the reported activity of compounds **120** and **122**–**124** [47].

A Western Australian *Axinella* sp. (Order: Axinellida; Family: Axinellidae) collected in the gulf of Exmouth was the source of the compounds herbindoles A (**126**), B (**127**) and C (**128**) (Figure 7) [48]. The structures of **126** to **128** were determined spectroscopically. The authors postulate that the biogenesis of the compounds is unlikely derived from tryptophan given the lack of substitution at position C-3 of the indole core. Compounds **126**–**128** exhibited cytotoxic activity against KB cells with an MIC of 5 µg/mL, >10 µg/mL and 10 µg/mL, respectively, and the combined extract also possessed significant fish feeding deterrent properties [48]. Two samples of *Trikentrion flabelliforme* (Order: Axinellida; Family: Raspailiidae) collected near Port Hedland yielded the new alkaloids trikentramides A–D (**129**–**132**) [49]. The planar structures and relative configurations of **129** to **132** were determined spectroscopically via comparison to prior literature reports [49]. Further evidence for the structures assigned was provided by quantum mechanical modelling and simulation of ^13^C NMR data as well as application of the DP4 algorithm pioneered by Goodman and co-workers [50]. Six new trikentrin-like natural products, (+)-*trans*-herbindole A (**133**) and trikentramides E–I (**134**–**138**) (Figure 7), have been recently reported from a sample of *Trikentrion flabelliforme* collected near Exmouth Gulf [51]. The relative and absolute configurations of **133** to **138** were determined by comparative analysis of optical rotation, computationally aided electronic circular dichroism spectroscopy (ECD) and chemical interconversion of the metabolites. The authors advance a plausible biosynthetic hypothesis for the formation of the trikentrin and herbindole classes of compounds beginning with the incorporation of a pyrrole-carboxylate thioester into a polyketide synthase. The authors also formulate an empirical mnemonic for the determination of the absolute stereochemistry of trikentrin and herbindole analogues dependant on the configuration of Me-C-8 [51].

**Figure 8:**
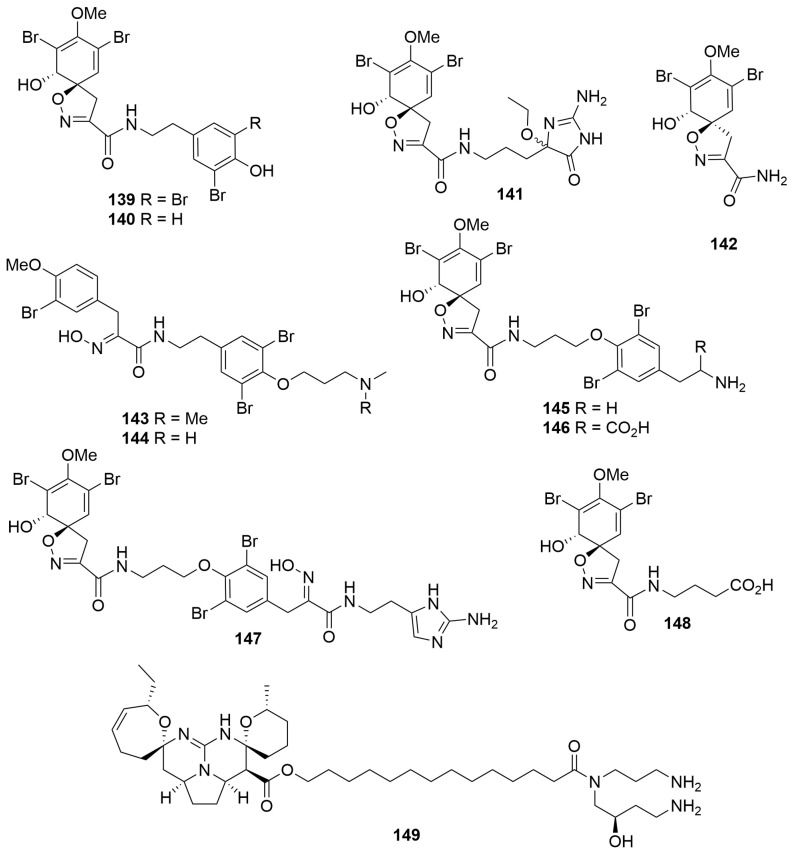
Compounds **139** to **149**.

Two new bromotyrosine alkaloids, pseudoceratinamides A (**139**) and B (**140**), as well as an artefact of extraction (**141**) and the enantiomer of a known compound (**148**), were isolated from a *Pseudoceratina* cf. *verrucosa* (Order: Verongida; Family: Pseudoceratinidae) collected off the Dampier Peninsula [52]. The sponge specimen also afforded the known compounds **142** to **147** (Figure 8). The planar and relative configurations of the compounds were determined spectroscopically. Absolute configurations of all the compounds were determined using specific rotation and ECD measurements. The authors note that the original depiction of araplysin I (**145**) depicted the wrong absolute configuration, despite no work being conducted towards the absolute configuration of the molecule. Promulgation of this mistake throughout the literature means that at least some of the compounds assigned in relation to araplysin I will have to be revised. More importantly, the authors note that the isolation of enantiomers of previously isolated compounds highlights the possibility of enantiodivergence in the biosynthesis of the bromotrosine spirooxazoline alkaloids at the epoxidative dearomatisation step [52]. All compounds isolated exhibited moderate activity against *Staphylococcus aureus* strains. Biological testing revealed that pseudoceratinamide A (**139**) and pseudoceratinamide B (**140**) exhibited significant activity (MIQ = 0.31 μg) against methicillin-sensitive *S. aureus*. Compounds **140**, **141**, **143**–**145** and **147** exhibited comparable activity to vancomycin (MIQ = 0.63 μg) against methicillin-resistant *S. aureus* [52]. 

A sample of *Monanchora viridis* (Order: Poecilosclerida; Family: Crambeidae) collected off Cape Mentelle in the southwest of the state yielded the known compound crambescidin 800 (**149**) (Figure 8). Compound **149** exhibited cytotoxic activity in a panel of breast cancer cell lines, with triple-negative breast cancer (TNBC) cells showing more significant differences in cell viability than immortalised fibroblasts. Additionally, **149** was shown to cause cell cycle arrest at G2/M phase in T11 and SUM159PT cells, as well as inhibit the phosphorylation of the Akt/mTOR, MAPK and NF-κB pathways, which are responsible for tumour relapse and metastasis [53]. 

#### 2.1.3. Polyketides

**Figure 9:**
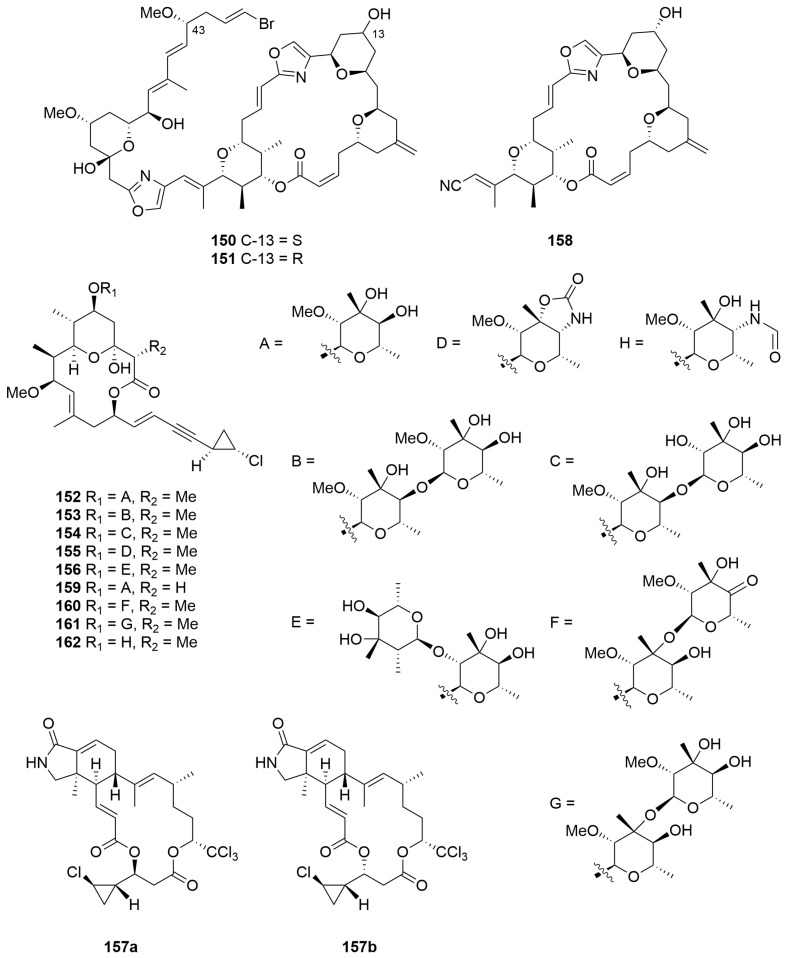
Compounds **150** to **162**.

Bioassay-guided fractionation of a *Phorbas* sp. (Order: Poecilosclerida; Family: Hymedismiidae) collected by hand using SCUBA near Muiron Island afforded the potent cytotoxins phorboxazoles A (**150**) and B (**151**), epimeric at position C-13, as pale yellow amorphous solids [7]. The planar structures of **150** and **151** were determined based on extensive COSY and HMBC experiments, and the relative configurations of all stereocentres on the macrolide hemisphere of the molecule were assigned with the aid of ROESY spectroscopy [7]. The authors note that assignment of the macrolide ring was facilitated by the conformational restrictions imposed by the three oxane rings and one oxazole ring present on the scaffold. Subsequent work established the relative configuration of the hemiketal ring system via synthesis of a model compound and the assignment of absolute configuration via Mosher’s ester analysis [54]. Finally, the stereochemistry of methoxy C-43 was assigned by chemical conversion to dimethyl methoxysuccinate and comparison to an authentic sample of the *R*-enantiomer by chiral GC-MS [55] (Figure 9). Phorboxazoles A (**150**) and B (**151**) exhibited antifungal properties against *Candida albicans*, as well as inducing cell growth inhibition across a spectrum of cancer cells (leukemia, CCRF-CEM, GI_50_ = 0.25 nM; HCT-116, GI_50_ = 0.44 nM), and displayed extraordinary cytostatic activity (mean panel GI_50_ < 7.9 pM) in the NCI 60 cancer cell line panel [7]. 

Re-examination of the same *Phorbas* sp. extracts using highly sensitive cryo-probe NMR experiments yielded two new chlorocyclopropane macrolides, phorbasides A (**152**) and B (**153**) (Figure 9) [56]. The assignment of absolute configuration was achieved via empirical comparison of ECD data obtained to that of synthesised model systems, taking advantage of the vibronic fine structure associated with an asymmetrically perturbed ene-yne chromophore [56]. Subsequent work afforded phorbasides C–E (**154**–**156**) [57], the highly chlorinated muironolide A (**157a**) [58], differing in the absolute configuration of the chloro-cyclopropane ring, along with the nitrile-bearing hemi-phorboxazole A (**158**) [59], and most recently, phorbaside F (**159**) [60] and phorbasides G–I (**160**–**162**) (Figure 9) [61]. The structure of muironolide A was subsequently revised to **157b** following total synthesis [62]. Biological evaluation of compounds **152**–**157** revealed modest cytotoxicity exhibited by the metabolites towards colon tumour cells (HCT-116; IC_50_ = 2–30 µM) with phorbaside C (**154**) exhibiting the most potent cytotoxic activity (IC_50_ = 2 µM). 

**Figure 10:**
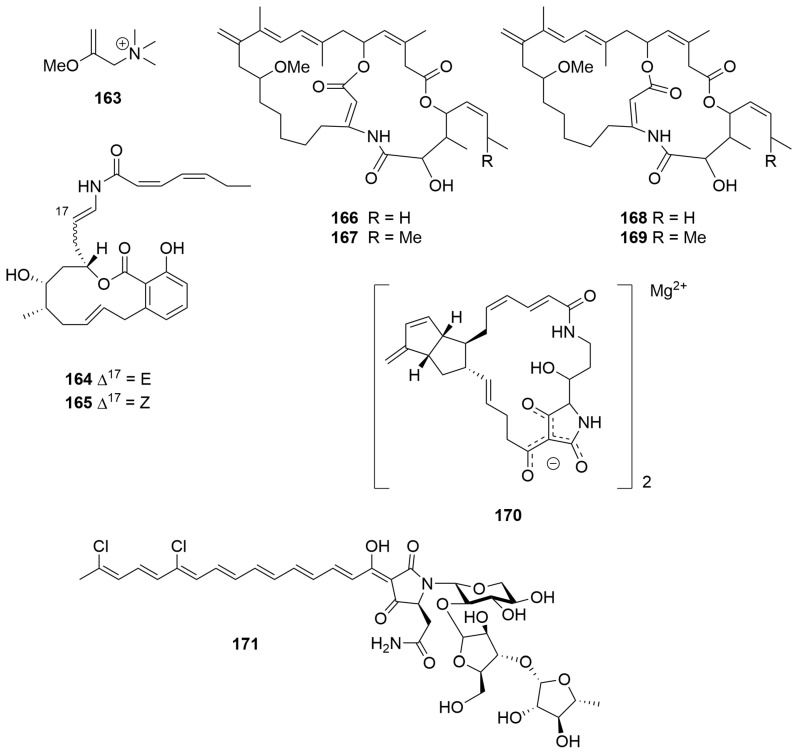
Compounds **163** to **171**.

Bioassay-guided fractionation of a *Raspailia* (raspalia) sp. (Order: Axinellida; Family: Raspailiidae) collected by trawl on the northern Rottnest Shelf afforded the known compounds phorboxazoles A and B (**150**, **151)** (Figure 9) as the principal nematocidal agents, as well as the known synthetic compound esmodil (**163**), isolated for the first time as a natural product [63]. The structure of **163** (Figure 10) was confirmed spectroscopically and via total synthesis. Biological testing revealed that **150** and **151** exhibited nematocidal activity against *Haemonchus contortus* (LD_99_ = 0.5 mg/mL and 1.1 mg/mL, respectively) [63]. 

Two novel macrolide antibiotics, salicylihalamides A (**164**) and B (**165**) (Figure 10), were reported from a *Haliclona* sp. (Order: Haplosclerida; Family: Chalinidae) collected off the coast of Rottnest Island. The compounds contain an unusual highly saturated ene-amide side chain [8]. Additional work has analysed the spatiotemporal distribution of the metabolites across species of *Haliclona* collected across the southwest of the state [64]. Compound **164** exhibited highly potent and specific cytotoxicity (mean panel GI_50_ = 15 nM) in the NCI 60 cell line human tumour screen. COMPARE pattern-recognition analysis revealed no significant correlations to the profiles of other known antitumour compounds, suggesting that the salicylihalamides represented a potentially important new class of compounds for antitumour lead optimisation [8]. Subsequent work determined the unprecedented mechanism of action of **164** and **165** via Vacuolar-ATPase inhibition [9].

A collection of two *Amphimedon* species (Order: Haplosclerida; Family: Niphatidae) collected during trawling operations in the Great Australian Bight afforded the novel macro-bicyclic lactones/lactams amphilactams A–D (**166**–**169**) (Figure 10) [65]. The planar structures of **166** to **169** were elucidated on the basis of extensive spectroscopic evidence and comparison to synthetic model compounds. The relative and absolute configurations of **166** to **169** remain unknown. Compounds **166** to **169** were isolated in sufficient amounts to quantify their in vitro LD_99_ activities against *Haemonchus contortus* as 7.5 µg/mL, 47 µg/mL, 8.5 µg/mL and 0.39 µg/mL, respectively [65]. 

Bioassay-guided fractionation of a *Geodia* sp. (Order: Tetractinellida; Family: Geodiidae) collected in the Great Australian Bight yielded a new macrocyclic polyketide lactam tetramic acid, as a magnesium salt **170** (Figure 10), as the sole agent responsible for the in vitro nematocidal activity of the extract [66]. The structure of geodin A (**170**) was determined spectroscopically. The magnesium content of the sample was determined by energy-dispersive spectroscopy and atomic absorption spectroscopy allowing the authors to deduce the presence of one unit of magnesium for every two units of tetramic acid [66]. Geodin A (**170**) exhibited potent in vitro nematocidal activity (LD_99_ = 1.0 µg/mL) [66]. 

A sample of *Manihinea lynbeazleyae* (Order: Tetactinellida; Family: Theonellidae) collected from Perth Canyon, Western Australia, yielded the known pigment aurantoside C (**171**) (Figure 10). Compound **171** exhibited specific cytotoxic activity against TNBC cells compared with non-TNBC cells [67]. 

#### 2.1.4. Fatty Acids

**Figure 11:**
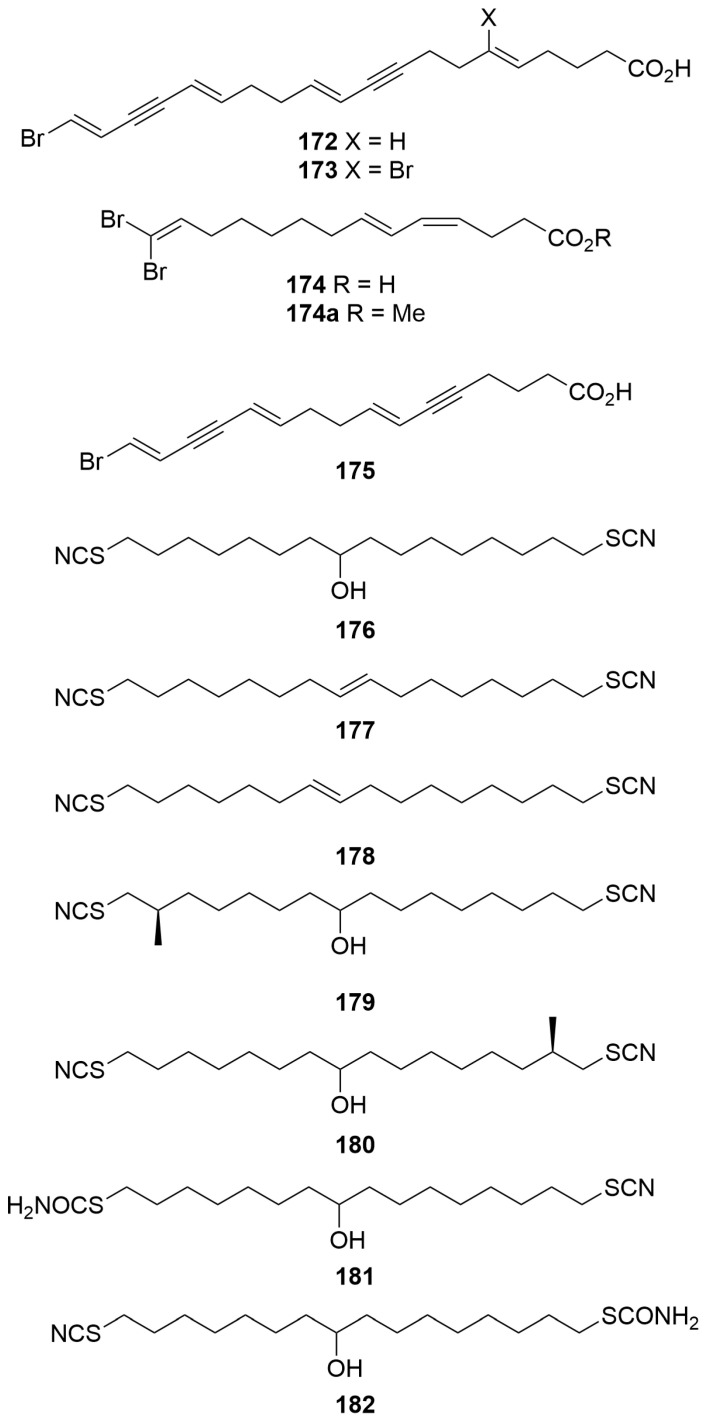
Compounds **172** to **182**.

A collection of three *Xestospongia* sp. (Order: Haplosclerida; Family: Petrosiidae) collected from Bennett shoal in the Exmouth gulf afforded the new polybrominated unsaturated fatty acids (5*E*,11*E*,15*E*,19*E*)-20-bromoeicosa-5,11,15,19-tetraene-9,17-diynoic acid (**172**), (5*Z*,11*E*,15*E*,19*E*)-6,20-dibromoeicosa-5,11,15,19-tetraene-9,17-diynoic acid (**173**) and (*Z*,*E*)-14,14-dibromo-4,6,13-tetradecatrienoate (**174**). Compound **174** was characterised as its methyl ester (**174a**) (Figure 11), and additional fractionation afforded the known carboxylic acid **175** [68]. The authors note the unusual carbon chain lengths present on the metabolites. Additionally, ribosomal RNA analysis of the sponge specimens indicated that up to 46% of the RNA present in the extracts was eubacterial in origin [68]. 

Bioassay-guided fractionation of an *Oceanapia* sp. (Order: Haplosclerida; Family: Phloeodictyidae) collected off the northern Rottnest Shelf afforded the novel dithiocyanates thiocyanatins A, B and C (**176**–**178**) (Figure 11) [69]. The structures of **176** to **178** were elucidated spectroscopically and confirmed in a seven-to-eight-step total synthesis starting from 8-bromooctanoic acid [69]. Re-analysis of the ethanolic sponge extract afforded thiocyanatins D_1_ and D_2_ (**177**, **180**) as an inseparable mixture and thiocyanatins E_1_ and E_2_ (**181**, **182**), also as an inseparable mixture, as well as a number of analogues tentatively identified by ^1^H NMR and LC-ESIMS [70]. The structures of the novel metabolites were elucidated with respect to the prior compounds **176** to **178** and via comparison to synthetic model compounds. The thiocyanatins exhibited potent nematocidal activity, and preliminary structure–activity relationship investigations confirmed the key characteristics of the thiocyanatin pharmacophore. Thiocyanatin A (**176**) exhibited potent nematocidal activity (LD_99_ = 1.3 µg/mL) against *Haemonchus contortus* [69]. 

**Figure 12:**
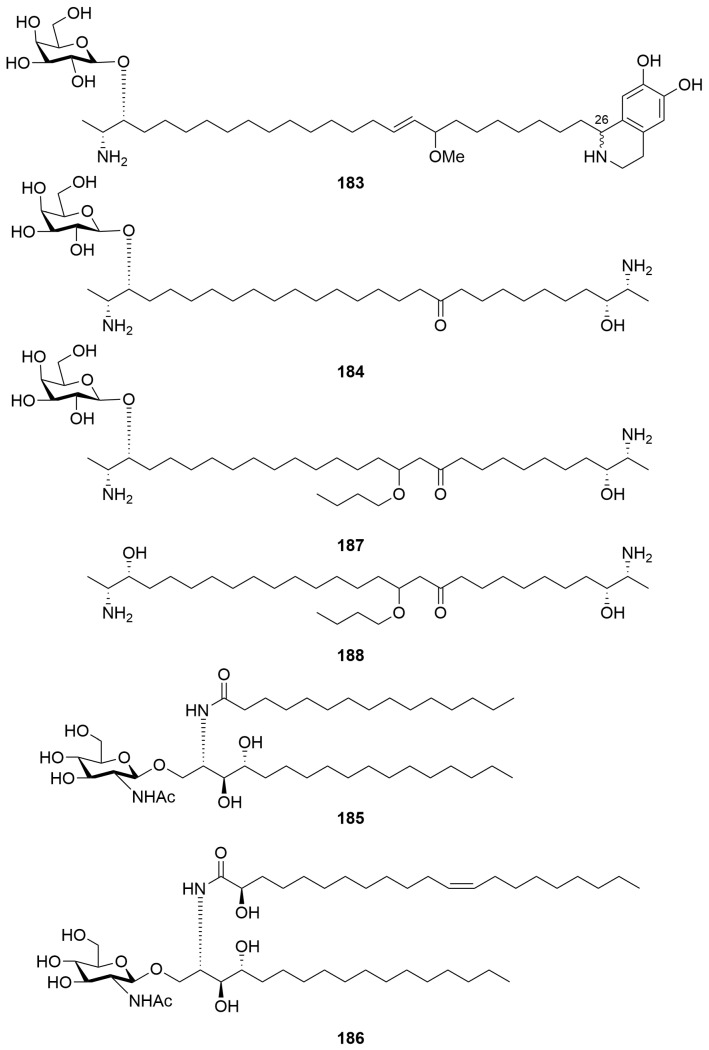
Compounds **183** to **188**.

An *Oceanapia* sp. collected by dredge off Scott Reef afforded the hybrid α,ω-bifunctionalised sphingoid tetrahydroisoquinoline β-glycoside oceanalin A (**183**), as well as the known compound rhizochalin (**184**) (Figure 12) [71]. The structure of oceanalin A was elucidated on the basis of ^1^H NMR and ^13^C NMR spectroscopy as well as chemical derivatisation. The authors conclude that, given the absence of optical rotation for the cleaved eastern hemisphere of the molecule, as well as the propensity of tetrahydroisoquinoline compounds to epimerise, compound **183** is likely a 1:1 mixture of epimers at the C-26 position [71]. A number of new cerebrosides of which two representative examples are depicted (**185**, **186**) were isolated from the same *Oceanapia* sp. collected off Scott Reef [72]. The cerebrosides were isolated as inseparable mixtures of compounds and assigned by NMR spectroscopy, MALDI-MS, chemical derivatisation and GC-MS [72]. Also from the same collection of *Oceanapia* sp. was isolated a ceramide fraction characterised by methanolysis and GC-MS analysis [73]. Most recently, the same collection of *Oceania* sp. afforded the new bolaampiphilic sphingoid bases rhizochalin B (**187**) and rhizochalinin B (**188**) (Figure 12) characterised by NMR spectroscopy as their peracetates [74]. The compounds contain an unusual butoxy group, which the authors note is uncommon in natural products. The ethanolic *Oceanapia* sp. extract exhibited antimicrobial activity against *Staphylococcus aureus*, *Bacillus subtilis*, *Escherichia coli* and *Candida albicans* and cytotoxic properties against the Erlich murine carcinoma. Metabolite **183** exhibited antifungal activity against *Candida glabrata* with an MIC of 30 µg/mL [71].

**Figure 13:**
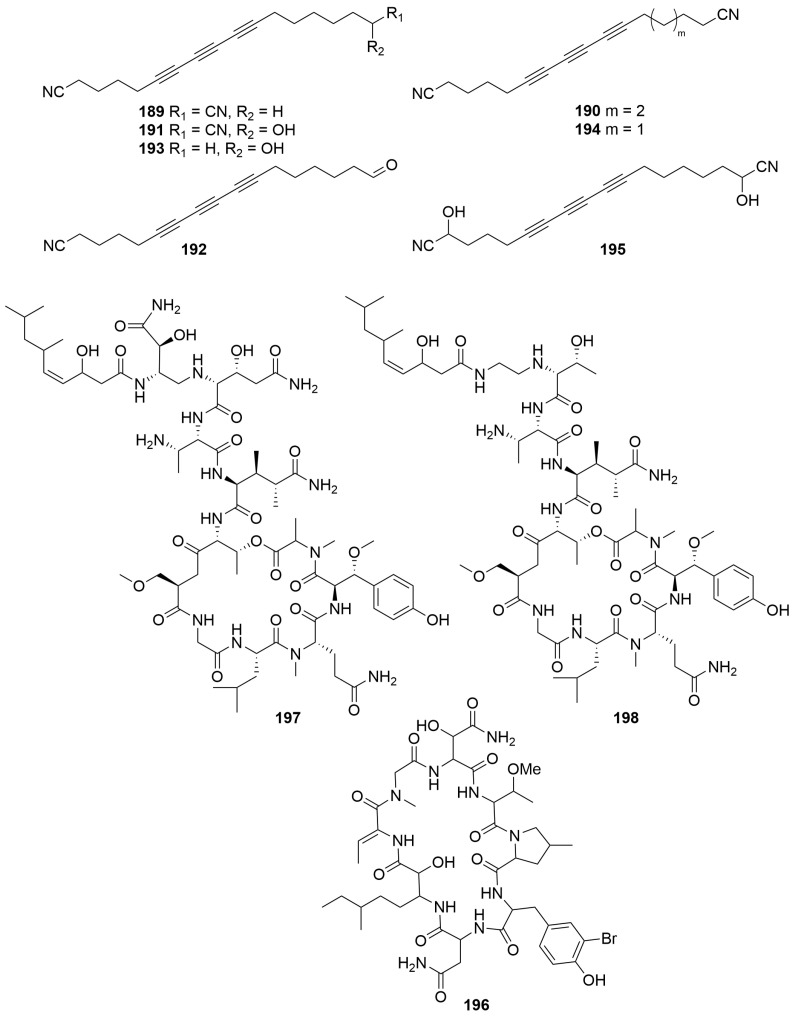
Compounds **189** to **196**.

A *Mycale* sp. (Order: Poecilosclerida; Family: Mycalidae) collected by benthic sled off the coast of Albany afforded seven novel polyacetylene nitriles: albanitriles A–G (**189**–**195**) (Figure 13) [75]. The compounds were characterised spectroscopically. Compounds **191** and **195** were isolated as racemic mixtures. Albanitrile C (**191**) exhibited mild toxicity towards *Bacillus subtilis* at 90 μM. Additional investigations revealed that compounds **189**–**192** exhibited activity against *Giardia duodenalis*, with albanitrile A (**189**) exhibiting activity at a minimum concentration of 12 μM, which was comparable to metronidazole used as the positive control. Compounds **189** to **191** also exhibited weak inhibition against *Tritrichomonas fetus*, on the order of 200 μM [75].

#### 2.1.5. Peptides

Bioassay-guided fractionation of a *Theonella* sp. (Order: Tetractinellida; Family: Theonellidae) collected by SCUBA near Perth, off Cape Vlamingh, afforded the cyclic octapeptide perthamide B (**196**) (Figure 13) [76]. The structure of compound **196** was elucidated spectroscopically; however, the relative and absolute configurations of the amino acid residues present remain unresolved. Compound **196** weakly inhibited binding of [^125^I]IL-1β to intact EL4.6.1 cells with an IC_50_ of 27.6 μM; however, the inhibition of binding could not be separated from the cytotoxic effects of **196** [76]. 

Two new HIV-inhibitory cyclic depsipetides, stellettapeptins A and B (**197**, **198**) (Figure 13), were isolated form a sample of *Stelletta* sp. (Order: Tetractinellida; Family: Ancorinidae) collected in the states northwest [77]. The compounds contain a number of unusual non-proteinogenic amino acids. The structures of **197** and **198** were determined spectroscopically and using Marfey’s analysis. Biological investigations revealed that compounds **197** and **198** exhibited infection-inhibitory activity of human T-lymphoblastoid cells by HIV-1RF with EC_50_ values of 23 nM and 27 nM, respectively [77].

#### 2.1.6. Miscellanea

**Figure 14:**
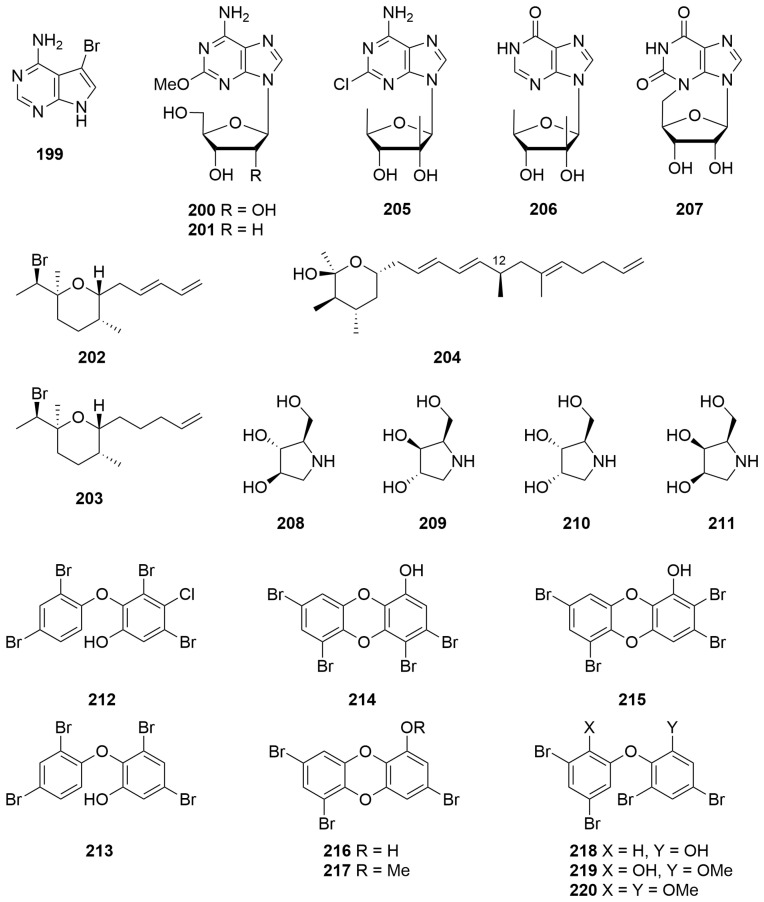
Compounds **197** to **220**.

Bioassay-guided fractionation of an *Echinodictyum* sp. (Order: Axinellida; Family: Raspailiidae) afforded 4-amino-5-bromopyrrolo [2,3-*d*]pyrimidine (**199**) (Figure 14) for the first time as a natural product. The identity of the metabolite was verified spectroscopically and via total synthesis [78]. Metabolite **199** exhibited potential as a bronchodilator [78]. 

The known nucleoside spongosine (**200**), previously reported by Bergmann et al. from *Cryptotethya crypta* and 2′-deoxyspongosine (**201**) (Figure 14), previously only reported as a synthetic, was isolated from a sponge of the Order Hadromerida (Tethyidae) collected by hand from Exmouth Gulf [79]. ^1^H and ^13^C NMR spectral data for both compounds were reported for the first time. 

Two new brominated tetrahydropyrans were obtained by preparative GC of a *Haliclona* sp. (Order: Haplosclerida; Family: Chalinidae) collected from Cosy Corner off the southwest coast of Western Australia [80]. The structures of (1′*R*,2S,2″*E*,5*R*,6*R*)-2-(1′-bromethyl)-2,5-dimethyl-6-(penta-2″,4″-dienyl)-tetrahydropyran (**202**) and (1′*R*,2*S*,5*R*,6*R*)-2-(1′-bromoethyl)-2,5-dimethyl-6-(pent-4″-enyl)-tetrahydropyran (**203**) (Figure 14) were determined spectroscopically and via chemical derivatisation. The absolute configuration of the compounds was determined using the Horeau method on an acyclic derivative [80]. Chemical investigations of a *Haliclona* sp. collected underneath an overhanging rocky substrate off Rottnest Island afforded **202** and **203**, as well as a novel tetrahydropyran, rottnestol (**203**), in low milligram quantity. The structure of **204** was elucidated on the basis of ^1^H, ^13^C, COSY, HSQC, HMBC and NOE NMR studies [81]. The stereochemistry at C-12 was subsequently determined by total synthesis [82].

A *Trachycladus laevispirulifer* (Order: Trachycladida; Family: Trachycladidae) collected in Exmouth Gulf afforded the unprecedented 2′-C-methyl-5′-deoxyribofuranosyl nucleosides Trachycladines A and B (**205**, **206**) (Figure 14) [83]. The structures of **205** and **206** were elucidated spectroscopically and by chemical derivatisation. Biological testing revealed that compound **205** exhibited cytotoxicity against several human cell lines, including leukaemia CCRF-CEM (IC_50_ = 0.4 µg/mL), colon tumour HCT-116 (IC_50_ = 0.9 µg/mL), breast tumour MCF-7 (IC_50_ = 0.2 µg/mL), MDAMB-435 (IC_50_ = 0.25 µg/mL) and MDA-N (IC50 0.1 µg/mL), but was inactive against yeasts (*Candida albicans*, *Saccharomyces carlsbergensis*) and bacteria (*Staphylococcus aureus*, *Escherichia coli* and *Pseudomonas aeruginosa*) in a disk diffusion assay at 200 µg/disk [83].

An *Erylus* sp. (Order: Geodiidae; Family: Erylinae; referred to in the original article as an *Eryus* sp.), collected west of Margaret River, yielded the first naturally occurring cyclo-nucleoside N-3,5′-cycloxanthosine (**207**) (Figure 14) [84]. The structure of the metabolite was elucidated spectroscopically and confirmed by total synthesis [84].

A sample of *Haliclona* sp. (Order: Haplosclerida; Family: Chalinidae) collected from Exmouth Gulf afforded 1,4-dideoxy-1,4-imino-D-arabinitol (**207**) (Figure 14), for the first time from a marine organism [85]. The identity of the compound was verified spectroscopically and by Marfey’s method. Investigation of a number of other sponges present in the same collection revealed the presence of **208** in a series of *Raspalia* sp. (Order: Axinellida; Family: Raspailiidae) as well as 1,4-dideoxy1,4-imino-D-xylitol (**209**), present both in a *Haliclona* sp. and *Raspalia* sp. Investigation of a *Cymbastela* sp. (Order: Axinellida; Family: Axinelllidae) revealed the presence of two additional isomers, identified tentatively as the diastereomers 1,4-dideoxy-1,4-imino-D-ribitol (**210**) and 1,4-dideoxy-1,4-imino-D-arabitol (**211**) (Figure 14) by Marfey’s analysis. Lack of authentic samples precluded the conclusive identification of **210** and **211** [85]. Compound **211** exhibited significant α-glycosidase inhibitory activity with an IC_50_ value of 0.16 μg/mL. Extracts containing compound **209** and the putative diastereomeric imino pentitols **210** and **211** exhibited significantly less α-glycosidase inhibitory activity [85].

Two polybrominated diphenol ethers, 3,5-dibromo-4-chloro-2-(2,4-dibromophenoxy)phenol (**212**) and 3,5-dibromo-2-(2,4-dibromophenoxy)phenol (**213**) (Figure 14), were isolated from an unidentified sponge collected by hand off the Rottnest Shelf. The structures of **212** and **213** were elucidated spectroscopically and confirmed by single-crystal X-ray diffraction [86].

A *Dysidea dendyi* (Order: Dictyoceratida; Family: Dysideidae) collected by hand from Scott Reef at a depth of 3 m afforded two new tetrabromodibenzo-*p*-dioxins: spongiadioxins A and B (**214**, **215**) (Figure 14) [87]. The structures of **214** and **215** were elucidated using a combination of 1D and 2D NMR spectroscopy. Additionally, the structure of **214** was verified by single-crystal X-ray diffraction of a methyl ether obtained from **214**. The structure of **215** was secured by chemical interconversion [87]. Re-investigation of the lipophilic sponge extracts afforded spongiadioxin C (**216**) and its methyl ether **217** as well as the related diphenyl ethers **218**–**220** [88]. The structures of the metabolites were determined spectroscopically and via semisynthesis. Biological evaluation revealed that compounds **214** and **215** exhibited cytotoxic activity against mouse Ehrlich carcinoma cells (ED_50_ = 29 and 15.5 µg/mL, respectively). In contrast, the LD_50_ for **214** and **215** in mice was determined to be more than 150 mg/kg [87]. Additional investigations revealed that **214**–**216** exhibited significant cell division inhibition for the fertilised eggs of the sea urchin *Strongylocentrotus intermedius* with an IC_50_ = 5.7 µM, 4.8 µM and 1.1 µM, respectively [88].

### 2.2. Cnidaria

**Figure 15:**
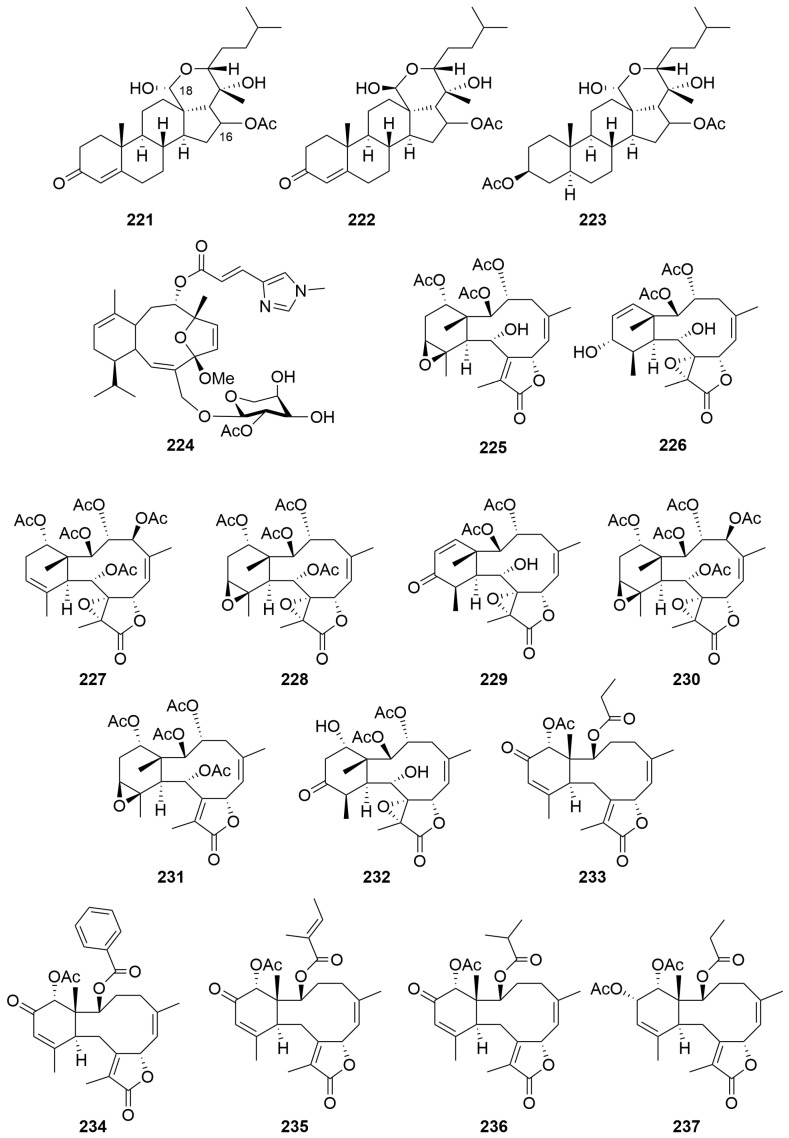
Compounds **221** to **237**.

A sample of *Ctenocella pectinata* (Order: Alcyonacea; Family: Ellisellidae), collected in Exmouth Bay, afforded three new sterols, pectinoacetals A–C (**221**–**223**) (Figure 15), isolated as their monoacetylated derivatives [89]. The underivatised natural products were found to undergo rapid interconversion of the C-18 hemiacetal chiral centre. The structures of the natural products were elucidated spectroscopically and by chemical derivatisation. The relative configuration of the stereocentre at C-16 could not be assigned conclusively by NOE spectroscopy [89].

Bioassay-guided fractionation of a rare Alcyonacean soft coral *Eleuthorobia* sp. (Order: Alcyonacea; Family: Alcyoniidae), found near Bennett’s Shoal, yielded the new diterpene glycoside eleutherobin (**224**) (Figure 15) [10]. The structure of **224** was assigned spectroscopically. Eleutherobin (**224**) exhibited significant specific cytotoxicity against a diverse panel of breast, renal, ovarian and lung cancer cell lines with an IC_50_ range of 10–15 nM. Compound **224** was found to stabilize microtubules by competing for the paclitaxel binding site on the microtubule polymer [10].

Fractionation of a freeze-dried specimen of *Briareum excavatum* (Order: Alcyonacea; Family: Briareidae), collected at Rowley Shoals, afforded the known diterpene (1R*,2R*,3R*,5*Z*,7S*,8(17)*Z*,10R*,11R*,12S*,14S*)-2,3,14-triacetoxy-11,12-epoxybriara-5,8(17)-dien-18-one (**225**), as well as the new briarane diterpenes excavatolides N–T (**226**–**232**) (Figure 15) [90]. The structures of **225** to **232** were assigned spectroscopically and by comparison to literature. The authors explain the unusual spectroscopic features presented by excavatolide T (**232**) by geometry optimisation of the proposed structure using density functional theory. Compounds **226**–**229** exhibited various levels of cytotoxic activity against P388 murine leukaemia, A549 human lung carcinoma, HT29 human colon carcinoma and MEL28 human melanoma cells [90].

A sea pen *Anthoptilum* cf. *kukenthali* (Order: Pennatulacea; Family: Anthoptilidae), collected by dredge at a depth of 267 m, northwest of Port Hedland, afforded five new briarane diterpenoids: the anthoptilides A–E (**233**–**237**) (Figure 15). The structure of anthoptilide A (**233**) was solved by single-crystal X-ray diffraction. Compounds **223**–**237** inhibited [^3^H]CPDPX binding to rat brain adenosine A1 receptors with IC_50_ values of 420 µM, 45 µM, 3.1 µM, 500 µM and 490 µM, respectively [91].

**Figure 16:**
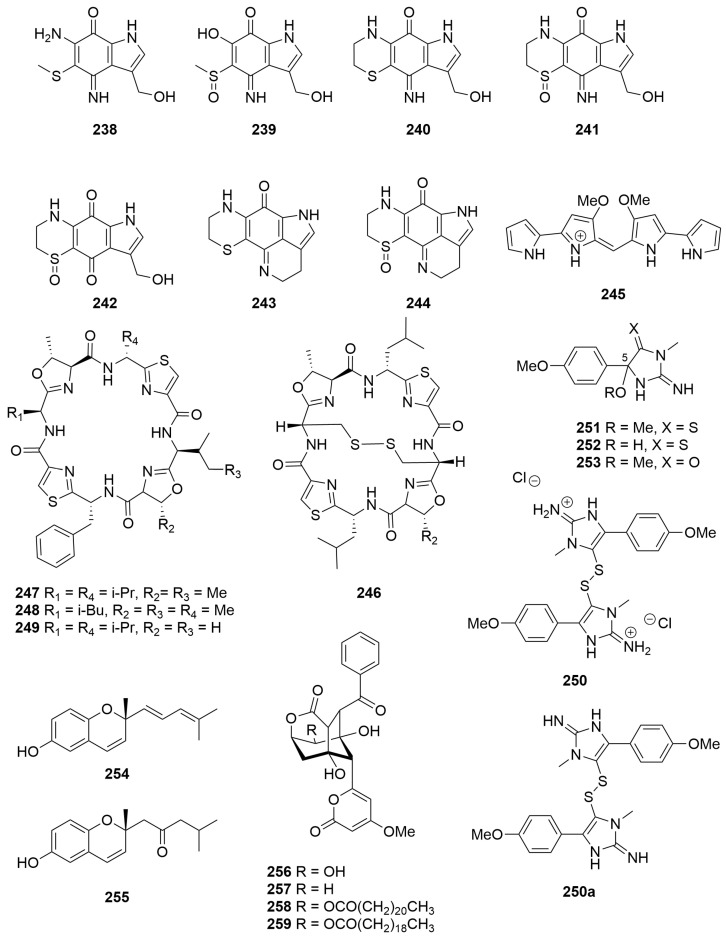
Compounds **238** to **259**.

A sample of the stinging Hydroid *Macrorhynchia philippina* (Order: Leptothecata; Family: Aglaopheniidae)*,* collected in the states northwest, afforded the novel pyrroloiminoquinone macrophilone A (**238**) (Figure 16) [92]. The structure of **238** was confirmed by total synthesis. Reinvestigation of the hydroid afforded the new metabolites, macrophilones B–G (**239**–**244**), the structures of which were elucidated using combined spectroscopic methods [93]. Compounds **238** to **244** are the first reported pyrroloiminoquinones from a marine Hydroid. The macrophilones **238**–**244** demonstrated inhibition of the enzymatic conjugation of SUMO to peptide substrates, and macrophilones A (**238**) and C (**240**) exhibited potent and specific cytotoxic activity in the NCI 60 cancer cell line panel [93]. Additionally, compound **238** showed sub-micromolar cytotoxicity towards lung adenocarcinoma cells [92].

### 2.3. Tunicata

Investigations of an ascidian collected in the Abrolhos Group afforded the deep blue tetra-pyrrole pigment **245** (Figure 16) [94]. The compound had previously been isolated from mutant strains of the Gram-negative bacterium *Serratia marcescens*, the structure of the pigment having been confirmed by total synthesis [95]. Microanalysis revealed that the ascidian pigment contained both chloride and bromide counter anions. Compound **245** exhibited an ability to increase the contractile force of guinea-pig ilea, with a dose-dependent increase evident [94].

Bioassay-guided fractionation of the colonial ascidian *Lissoclinum patella* (Order: Aplousobranchia; Family: Didemnidae), collected from the Montebello Archipelago, afforded three known cyclic peptides, ulithiacyclamide (**246**), lissoclinamide (**247**) and patellamide B (**248**), as well as the novel patellamide F (**249**) (Figure 16) [96]. The absolute configuration of pattelamide F (**249**) was confirmed by Marfey’s analysis. Patellamide B (**248**), patellamide F (**249**) and ulithiacyclamide (**246**) exhibited modest general cytotoxicity in the NCI 60 cell line human tumour screen with LC_50_ values of 48 µM, 13 µM and 3 µM, respectively [96].

A new dimeric disulfide alkaloid, polycarpine (**250**) (Figure 16), was isolated as its dihydrochloride salt from the extracts of the ascidian *Polycarpa clavata* (Order: Stolidobranchia; Family: Styelidae) [97]. Purification of the metabolite on silica afforded the free base **250a** which readily decomposed to the monomeric products **251** to **253**, arising from nucleophilic addition of water or methanol to position C-5 of the imidazole ring followed by cleavage of the disulfide bond [97]. Dissection of the organism into anatomical parts and fresh extraction in MeOH followed by immediate acquisition of NMR spectra demonstrated that compound **249** was the sole natural product and that it was located entirely in the organism’s branchial sac. Polycarpine dihydrochloride (**250**) exhibited cytotoxic activity against the human colon tumour cell line HCT-116 at 0.9 μg/mL [97].

Fresh specimens of a tunicate tentatively identified as *Aplidium solidum* (Order: Aplousobranchia; Family: Polyclinidae), collected in the Great Australian Bight, afforded the new chromenols (*R*)-2-methyl-2-(4-methylpenta-1,3-dienyl)-2*H*-chromen-6-ol (**254**) and 1-[(*R*)-6-hydroxy-2-methyl-2*H*-chromen-2-yl]-4-methylpentan-2-one (**255**) (Figure 16) [98]. The structures of **254** and **255** were elucidated spectroscopically. The absolute configuration of the metabolites was determined by hydrogenation and ozonolysis with acid work-up followed by comparison to the optical rotation of the known compound (*R*)-4,8-dimethylnonan-4-olide [98].

Chemical investigation of the brown encrusting ascidian *Didemnum* sp. (Order: Aplousobranchia; Family: Didemnidae), collected by hand using SCUBA, near Exmouth, afforded the known bacterial metabolite enterocin (**256**) as well as 5-deoxyenterocin (**257**), previously reported in a Japanese patent albeit with no spectroscopic data reported. Reversed-phase HPLC of the lipohilic fractions afforded the novel esters enterocin-5-behenate (**258**) and entorocin-5-arachidate (**259**) (Figure 16), assigned spectroscopically [99]. 

**Figure 17:**
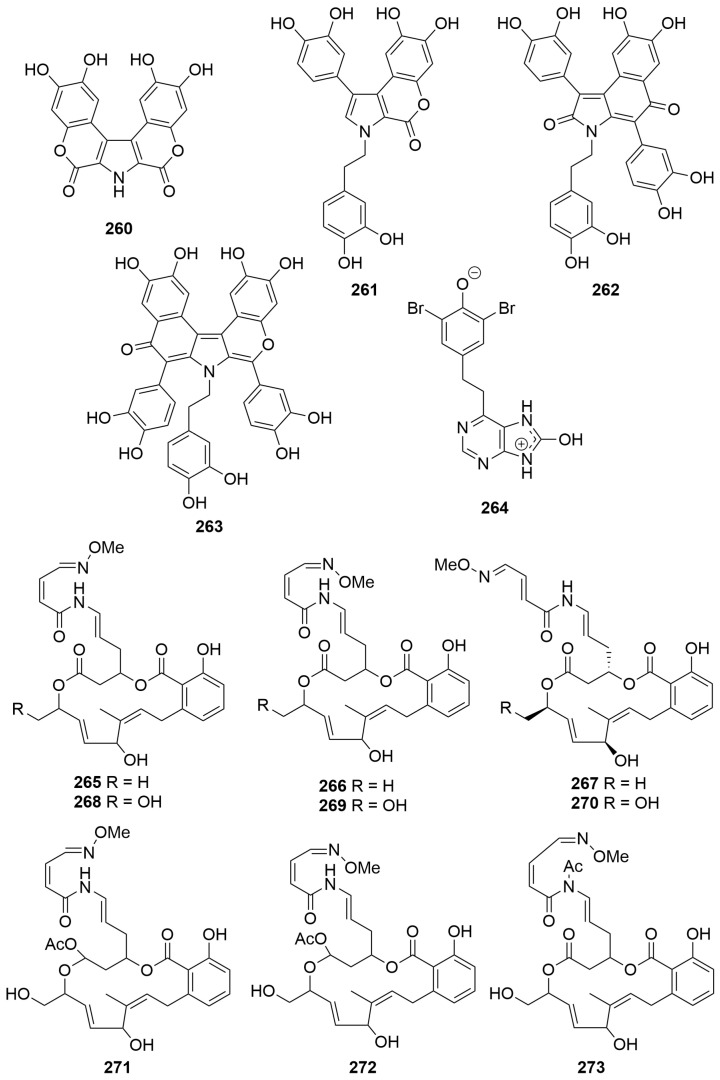
Compounds **260** to **273**.

An undescribed ascidian *Didemnum* sp. (Order: Aplousobranchia; Family: Didemnidae), collected near Ningaloo Reef, yielded four new aromatic alkaloids ningalins A–D (**260**–**263**) (Figure 17), assigned structures **260** to **263** on the basis of spectroscopic analysis [100]. The authors propose a biosynthetic route to compounds **260** to **263** deriving from repeat condensation of DOPA. The absence of optical rotation in compounds **262** and **264** indicated that they are likely present as mixtures of racemates arising from rotamerism and helicity of the scaffolds [100].

Fractionation of an ascidian *Aplidiopsis* sp. (Order: Enterogona; Family: Polyclinidae) collected near Ningaloo Reef afforded the zwitterionic hydroxyadenine aplydiamine (**264**) (Figure 17). The structure of **264** was elucidated spectroscopically and by chemical derivatisation. The assignment of **264** as a zwitterion was based on HMBC correlations in d_6_-DMSO and the observed NOE correlation between all three exchangeable protons [101]. 

Two new cytotoxic macrolides, lobatamides A and B (**265** and **266**) (Figure 17), structurally related to the salicylihalamide class of macrolides, were isolated following bioassay-guided fractionation of an *Aplidium lobatum* (Order: Aplousobranchia; Family: Polyclinidae) [102]. Critical evidence for the structures of **265** and **266** was provided by analysis of the FAB-MS data. Re-investigation of the *A. lobatum* afforded the lobatamides C–F (**267**–**270**), the structures of which were elucidated spectroscopically [103]. The authors report the isolation of compounds **265** to **270** from three different shallow-water collections of Australian *A. lobatum*, an *Aplidium* sp. collected during a trawling expedition at the Great Australian Bight and finally from an unidentified, shallow-water collection of a Philippine tunicate. The authors note the spectral similarities between the lobatamides A–D (**265**–**268**) and the aplidites A–D, isolated from a Great Australian Bight *Aplidum* sp. [104], and propose revising the structures of the latter compounds to structures **265**–**268**, respectively. The authors also propose revising the structures of the related aplidites E-G to structures **271** to **273** and renaming the natural products lobatamides G–I, respectively [103]. Given the reported isolation of lobatamide A from a species of terrestrial pseudomonad and the isolation of the related salicylihalamide macrolides from a marine sponge, the authors postulate a likely microbial origin for the family of compounds [103]. The relative and absolute configuration of lobatamide C (**267**) was subsequently confirmed following total synthesis of **267** by the Porco group [105]. Biological testing revealed that the lobatamides A–D (**265**–**268**) exhibited approximately equipotent specific cytotoxicity in the NCI 60 cell line human tumour screen (mean panel GI_50′s_ ~1.6 nM). COMPARE pattern-recognition analysis revealed no significant correlations to the profiles of other known antitumour compounds, suggesting that compounds **265**–**268** may act by a novel mechanism of action. The differential cytotoxicity profiles of the compounds **265**–**268** did, however, show high (>0.7) COMPARE correlations among themselves, as well as with the salicylihalamides A and B (**164**, **165**) isolated from the marine sponge *Haliclona* sp. The authors remark that the result is not surprising, given the structural similarities between the two compound families [103].

### 2.4. Echinodermata

**Figure 18:**
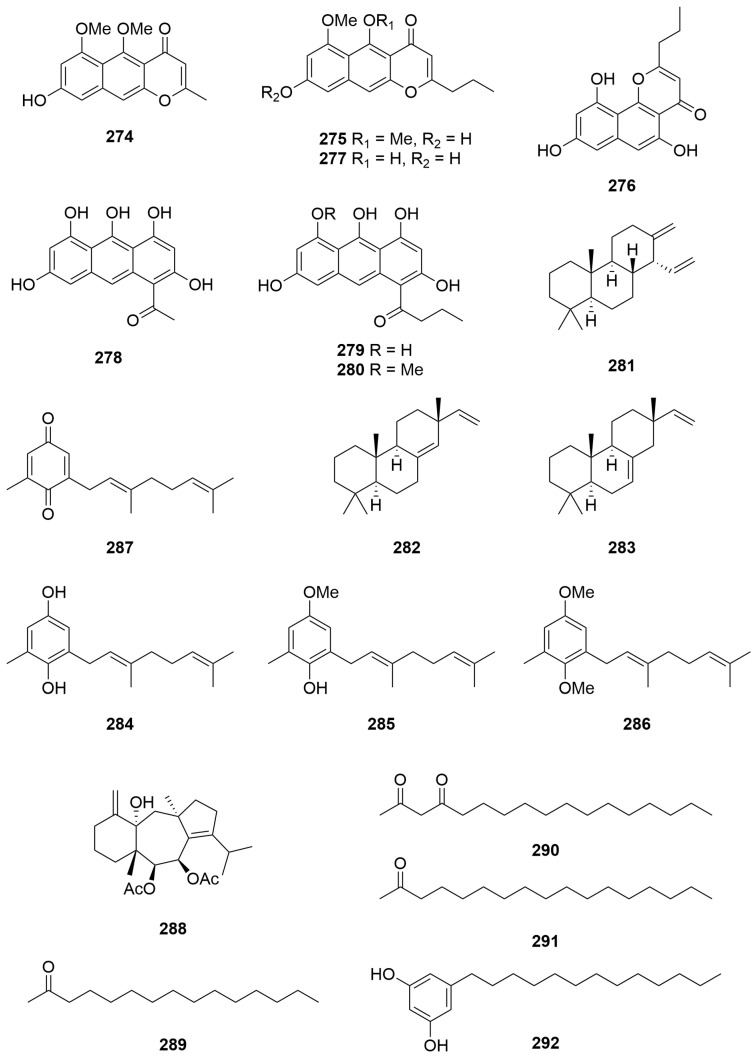
Compounds **274** to **292**.

Investigation of the pigments of the crinoid *Comantheria briareus* (Bell) (Order: Comatulida; Family: Comatulidae) afforded the known naphthapyrone polyketides comantherin (**273**), neocomantherin (**275**) and comaparvin (**276**) as well as a novel pigment 5,8-dihydroxy-6-methoxy-2-propyl-4*H*-naphtho [2,3-*b*]pyran-4-one (**277**) (Figure 18). Isolation of the pigments present in *Comatula solaris* (Order: Comatulida; Family: Comatulidae) afforded the known compounds rhodolamprometrin (**278**) and rhodocomatulin-6,8-dimethyl ether (**279**). Chemical investigation of *Comatula rotolaria* afforded compounds **278** and **279** as well as rhodocomatulin-6-methyl ether (**280**) [106]. The echinoderm specimens analysed were all collected in the nets of prawn trawlers operating out of Carnarvon. 

### 2.5. Plantae

A new cleistanthene diterpene hydrocarbon (**281**) was isolated from the leaves of *Amphibolis antartica* (Order: Alismatales; Family: Cymodoceaceae) collected from Shark Bay [107]. The structure of **281** was assigned spectroscopically. Chemical instability of the compound prevented degradative analysis. Samples of *A. antartica* collected near Perth contained **281**, as well as the known derivatives sandaracopimaradiene (**282**) and isopunaradiene (**283**) (Figure 18), identified by GC-MS analysis. Analysis of individual specimens collected from Shark Bay by GC-MS revealed that the *n*-hydrocarbon content diminishes with maturity of the specimen, whereas concentration of **281** increases with leaf age [107]. 

### 2.6. Ochrophyta

Chromatography of the CH_2_Cl_2_ extracts of the brown algae *Cystophora* sp. (Order: Fucales; Family: Sargassaceae) collected from the wave-swept rock platforms of Cosy Corner, southwest WA, afforded three new isoprenoid dihydroquinones derived from geranyltoluquinol. The structures of the compounds were deduced as **284** to **286** (Figure 18) by ^1^H and ^13^C NMR spectroscopy and chemical interconversion [108]. Earlier isolation attempts had led to isolation of benzoquinone **287**. The authors conclude that **287** is not a genuine natural product as acetylation of the *Cystophora* sp. crude extract led to the isolation of a diacetylated derivative of **284** and the observation that benzoquinone **287** was not present [108]. 

Isolation of the major lipophilic metabolite from a sample of *Dictyota furcellata* (Order: Dictyotales; Family: Dictyotaceae) collected from Cape Peron, Shark Bay, afforded the new dolastane diterpenoid (6*S*,7*R*,14*S*)-6,7-diacetoxydolasta-1(1*S*),8-dien-14-ol (**288**) [109]. The structure of **288** was deduced spectroscopically and confirmed by single-crystal X-ray diffraction.

Three acetogenin metabolites **289**–**291** and an alkyl resorcinol **292** (Figure 18) were isolated from the brown algae *Caulocystis cephalornithos* (Order: Fucales; Family: Cystoseiraceae) collected from Beacon Island, Wallabi Group. The structures of the metabolites were assigned as pentadecan-2-one (**289**), heptadecan-2,4-dione (**290**), heptadecan-2-one (**291**) and 5-tridecylresorcinol (**292**) on the basis of GC-MS and spectroscopic analysis [110].

**Figure 19:**
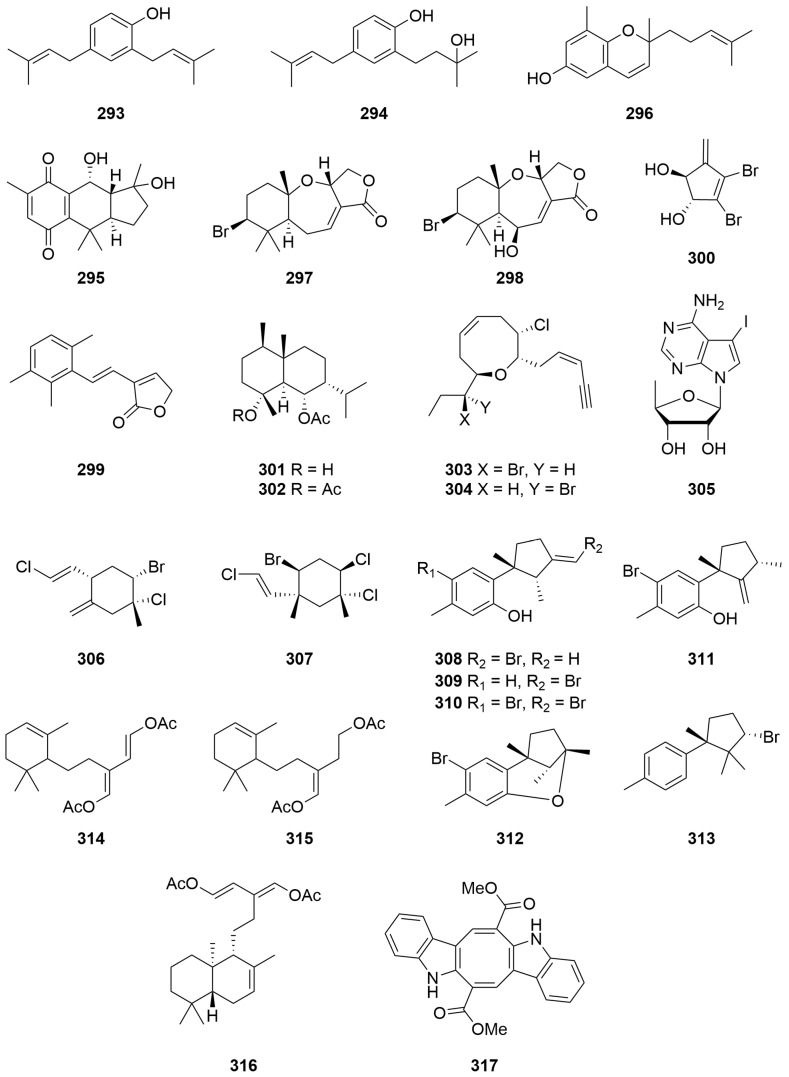
Compounds **293** to **317**.

A sample of the brown alga *Encyothalia cliftonii* (Harvey) (Order: Sporochnales; Family: Sporochnaceae) afforded two bisprenylated phenols: 2,4-bis(3-methylbut-2-enyl)phenol (**293**), reported previously from *Perithalia caudata* and 2-(3-hydroxy-3-methylbutyl),4-(3-methylbut-2-enyl) phenol (**294**) (Figure 19) [111], the structures of which were solved spectroscopically and by chemical derivatisation. Biological testing indicated that 2,4-bis(3-methylbut-2-enyl)phenol (**293**) exhibited significant feeding deterrence towards the herbivorous sea urchin *Tripneustes esculentus* [111].

Chemical investigation of the brown alga *Cystophora harveyi* (Order: Fucales; Family: Sargassaceae), collected by SCUBA from the first bay to the east of the Cape Leeuwin lighthouse, yielded the new linearly fused tricyclic compound pycnanthaquinone C (**295**), the structure of which was elucidated spectroscopically [112]. Fractionation of the crude extract also afforded the known compounds atractylochromene (**296**), (2′*E*)-2-(3′,7′-dimethylocta-2′,6′-dienyl)-4-hydroxy-1-methoxy-6-methylbenzene (**285**), (2′*E*)-1,4-dimethoxy-(3′,7′-dimethylocta-2′,6′-dienyl)-6-methylbenzene (**286**) and (2′*E*)-2-(3′,7′-dimethylocta-2′,6′-dienyl)-6-methyl-2,5-cyclohexadiene-1,4-dione (**287**) (Figure 19). Atractylochromene (**296**) was reported for the first time from a marine organism. The authors provide a biosynthetic scheme for the formation of compounds **285** to **287** and **295** to **296**. In addition, the authors note that compound **296** has previously been reported as an effective anti-inflammatory agent [112].

### 2.7. Rhodophyta

A sample of *Laurencia filiformis* (Order: Ceramiales; Family: Rhodomelaceae) collected from Point Peron yielded the sesquiterpene metabolites aplysisistatin (**297**), previously isolated from the sea hare, *Aplysia angasi*, as well as 6β-hydroxyaplysistatin (**298**) (Figure 19) [113]. The structures of **297** and **298** were assigned crystallographically. A chance observation led to the discovery that thermal rearrangement of 6β-hydroxyaplysistatin (**298**) afforded one major decomposition product **299** involving the formal loss of one unit of HBr and two units of water. The structure of the thermolysis product **299** was confirmed by Capon and Ghisalberti in a five-step total synthesis [114]. 

Chemical investigation of the red alga *Vidalia spiralis* (Order: Ceramiales; Family: Rhodomelaceae) collected at Yanchep yielded the new halogenated diol 3,4-dibromo-5-methylenecyclopent-3-ene-1,2-diol (**300**) (Figure 19) as a fine crystalline powder [115]. The structure of **300** was determined spectroscopically and by chemical derivatisation. Attempts to monoacetylate the diol failed, precluding the use of Horeau’s method to determine the absolute configuration of the natural product. The *Vidalia spiralis* crude dichloromethane extract exhibited hypotensive activity, and the crude methanol extract exhibited stimulant activity. Neither of these activities was evident, however, in the purified compound [115]. 

Fractionation of the lipophilic extracts of a sample of the red algae *Laurencia filiformis* (Order: Ceramiales; Family: Rhodomelaceae) yielded the novel brominated eudesmane sesquiterpenes austradiol acetatete (**301**) and austradiol diacetate (**302**) as well as the known *cis*-dihydrorhodophytin (**303**) and *cis*-epidihydrorhodophytin (**304**) (Figure 19) [116]. The structures of **301** and **302** were elucidated spectroscopically and by chemical derivatisation. Evidence for the proposed twist-boat conformation of austradiol acetate (**301**) was provided by complexation with a europium chemical shift reagent [116]. 

Bioassay-guided fractionation of a methanolic extract of the red alga *Hypnea valendiae* (Order: Gigartinales; Family: Cystocloniaceae), collected at Quobba Lagoon, returned 4-amino-7-(5′-deoxyribose-1′β-yl)-5-iodopyrrolo [2,3-*d*]pyrimidine (**305**) (Figure 19) as the principle active metabolite, the compound exhibited an ability to induce muscle relaxation and hypothermia [78]. A minor metabolite isolated from a subsequent extraction of *Hypnea valendiae* was tentatively assigned as the α-1′ isomer **305** by ^1^H NMR. Paucity of material and the requirement for bioassay prevented further structural validation [78].

Direct sublimation of the methylene chloride soluble extract of *Plocamium mertensii* (Order: Plocamiales; Family: Plocamiaceae), collected at Carnac Island, yielded (1*R*,2*S*,4*S*,1′*E*)-2-bromo-l-chloro-4-(2′-chloroetheny1)-1-methyl-5-methylenecyclohexane (**306**). The structure of **306** was solved by single-crystal X-ray diffraction [117]. An unidentified *Plocamium* species collected from the beach wash on Rottnest Island yielded a small quantity of crystalline (1*R*,2*S*,4*R*,5*R*,1′*E*)-4-bromo-l,2-dichloro-5-(2′-chloroetheny1)-l,5-dimethylcyclohexane (**307**) (Figure 19), the structure of which was also solved by single-crystal X-ray diffraction [117]. (1*R*,2*S*,4*S*,1′*E*)-2-Bromo-1-chloro-4-(2′-chloroethenyl)-1-methyl-5-methylenecyclohexane (**306**) exhibited unusual biological activity. Apart from weak cytostatic and antibacterial activity, the authors note that the compound produced a ‘spastic’ syndrome in mice which persisted for several days but was, however, reversible [117].

Samples of *Laurencia filimformis* f. *heteroclada* (Order: Ceramiales; Family: Rhodomelaceae) were collected from four sites along the West Australian coast. All of the samples were found to afford laurene sesquiterpene metabolites [118]. From the sample collected from Hamelin Bay was isolated allo-laurentirol (**308**). The sample from Lancelin afforded laurenisol (**309**), and the sample collected from Cottesloe Beach afforded bromolaurenisol (**310**). Fractionation of the sample collected at Shoalwater Bay, Rockingham, yielded laurenisol (**309**), bromolaurenisol (**310**), isolaurentirol (**311**), filiformin (**312**) and (−)-α-bromocuparene (**313**) (Figure 19) [118].

### 2.8. Chlorophyta

Two sesquiterpene metabolites were isolated from a specimen of *Caulerpa flexilis* var. *muelleri* (Order: Bryopsidales; Family: Caulerpaceae) collected from Cosy Corner. The structure of the metabolites was confirmed as (1*E*,3*E*)-2-[2′-(2″,6″,6″-trimethylcyclohex-2″-enyl)ethyl]buta-1,3-diene-I,4-diyl diacetate (**314**) and (2*E*)-3-formyl-5-(2′,6′,6′-trimethylcyclohex-2′-enyl)pent-2-enyl acetate (**315**) (Figure 19) using combined spectroscopic information [119]. The geometry of the trisubstituted double bond present on **314** was inferred from NOE experiments; however, the authors caution against conclusive assignment [119].

A sample of *Caulerpa trifaria* (Order: Bryopsidales; Family: Caulerpaceae) collected at Point Peron afforded the new sesquiterpene metabolite **316**, the structure of which was deduced spectroscopically. The absolute configuration of the compound remains unknown. Samples of *C. brownii*, *C. pexilis*, *C. peltata* and *C. racemosa* also collected from Point Peron failed to yield **316**. However C. peltata and *C. racemosa* afforded caulerpin (**317**) in low yield as red-plate crystals [120].

### 2.9. Cyanophyta

**Figure 20:**
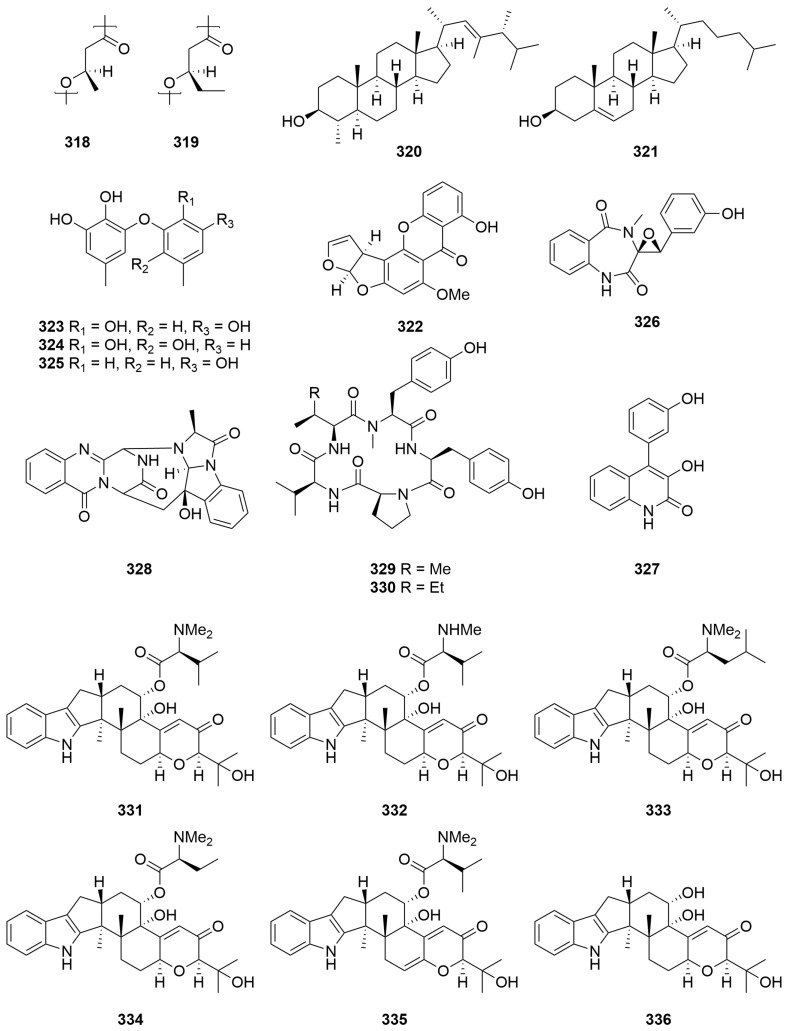
Compounds **318** to **336**.

A sample of the freshwater cyanobacterium *Aphanothece* sp. (Order: Chroococcales; Family: Aphanothececeae) collected from Lake Joondalup afforded a polyester mixture composed of (*R*)-3-hydroxybutanoic acid (**318**) and (*R*)-3-hydroxypentanoic acid (**319**) in an approximate 2:1 ratio (Figure 20) [121]. The nature of the polymer was determined by hydrolysis, followed by spectral, chiroptical and GC-MS characterisation. Chemical analysis of *Microcoleus* sp. (Order: Oscillatoriales; Family: Microcoleaceae), *Lyngbya aestuani* (Order: Oscillatoriales; Family: Oscillatoriaceae), *Murocoleur (Microcoleus) chthonoplastes* and *Entophysalis deusta* (Order: Chroococcales; Family: Entophysalidaceae) stromatolite cyanobacterial mats collected from Shark Bay revealed the presence of similar polyesters to those found in *Aphanothece* sp. collected from Lake Joondalup [121].

### 2.10. Dinoflagelatta

Capillary GC-MS analysis of four closely related species of marine dinoflagellate identified dinosterol (**320**) (Figure 20) as the major sterol constituent of *Prorocentrum balticum* (Order: Prorocentrales; Family: Prorocentraceae) and *Prorocentrum minimum* [122]. Cholesterol (**321**) (Figure 20) was found to be the major constituent of *Prorocentrum micans* and *Prorocentrum mexicanum* [122]. Other steroid components were identified and annotated by GC-MS for all four species. The authors propose that the similarity of steroidal fractions from members of the same species grown in different laboratories suggests a strong genetic, rather than environmental, influence on the steroidal composition of such species and that the steroidal profiles reported may be used to delineate the species chemotaxonomically [122].

### 2.11. Fungi

A marine-derived *Aspergillus versicolor* (MST-MF495) (Order: Eurotiales; Family: Trichocomaceae) isolated from a sample of beach sand collected at Cottesloe afforded the known compounds sterigmatocystin (**322**), violaceol I (**323**), violaceol II (**324**), diorcinol (**325**), (−)-cyclopenol (**326**) and viridicatol (**327**), as well as the novel alkaloid cottoquinazoline A to which was assigned the partial relative stereostructure **328**, and the two novel cyclic pentapeptides cotteslosins A and B (**329**, **330**) (Figure 20) [123]. The structures of **328** to **330** were assigned spectroscopically and via the modified C3-Marfey’s Analysis. Violaceol I (**323**), violaceol II (**324**) and diorcinol (**325**) exhibited antibacterial properties. Additional biological testing indicated that the novel peptide **329** showed weak cytotoxic activity against human melanoma (MM418c5, EC_50_ = 66 µg/mL), prostate (DU145, EC_50_ = 90 µg/mL) and breast (T47D, EC_50_ = 94 µg/mL) cancer cell lines. Cotteslosin B (**330**) was reported to exhibit weak cytotoxic activity [123].

Recently, cultivation of a marine-derived *Aspergillus noonimiae* collected in waters near Perth afforded the indolic diterpenes noonindoles A–F (**331**–**336**) (Figure 20) as well as a number of minor metabolites putatively assigned via tandem MS analysis. Structures of the major compounds were assigned following detailed spectroscopic analysis and single-crystal X-ray diffraction. Testing of the metabolites against a panel of microorganisms revealed that the compounds were essentially devoid of biological activity, with the exception of mild antifungal activity displayed by **331** against *Candida albicans* [124].

### 2.12. Arsenic Metabolism in the Marine Food Web

**Figure 21:**
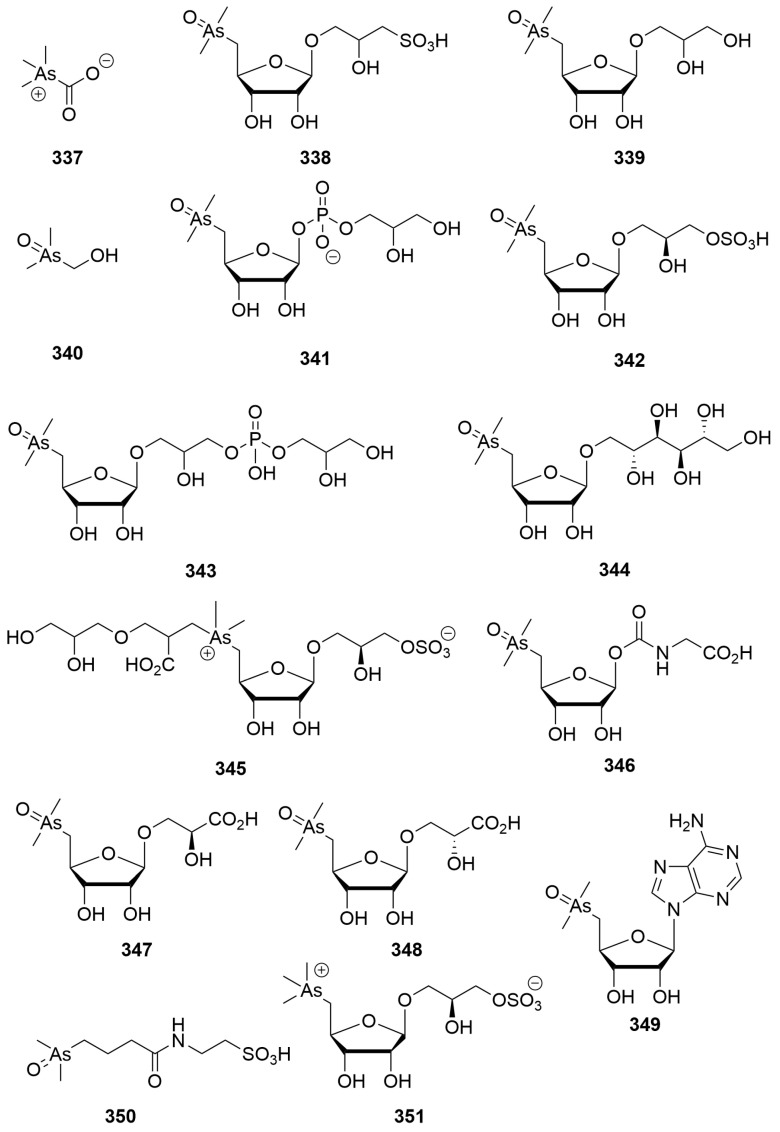
Compounds **337** to **351**.

Vapour generation atomic absorption spectrometry guided fractionation of the commercially important western rock lobster, *Panulirus cygnus* (George) (Order: Decapoda; Family: Paluniridae), afforded arsenobetaine (**337**) (Figure 21) as the principal arsenic-containing metabolic constituent [125]. The structure of **337** was elucidated crystallographically and confirmed by total synthesis [125].

Investigation of the arsenical constituents of the brown kelp *Ecklonia radiata* (Order: Laminariales; Family: Lessoniaceae) afforded the metabolites 2-hydroxy-3-sulphopropyl-*S*-deoxy-5-(dimethylarsenoso)furanoside (**338**) and a 2,3-dihydroxypropyl-*S*-deoxy-*S*-(dimethylarsenoso)furanoside (**339**) (Figure 21) [126]. The authors propose that the compounds may act as intermediates between arsenate present in seawater and arsenobetaine present at higher trophic levels [126]. Key evidence for this proposal came from experiments investigating the anaerobic decomposition *Ecklonia radiata*, affording the new compound dimethyloxarsylethanol (**340**) [127]. Additional investigations of *Ecklonia radiata* yielded the minor metabolite 3-glycerophosphoryl-2-hydroxy-I-[5-deoxy-5-(dimethylarsinoyl)-β-ribofuranosyloxy]propane (**341**). The structure of this metabolite was elucidated principally by NMR spectroscopy [128].

Compound **342**, as well as (2*S*)-3-[5-deoxy-5-(dimethylarsinoyl)-β-D-ribofuranosyloxy]-2-hydroxypropyl hydrogen sulfate (**343**), was isolated from the kidney of the giant clam, *Tridacna maxima* (Order: Cardiida; Family: Cardiidae), collected from Shark Bay [129]. The structure of **343** was solved using single-crystal X-ray diffraction. Targeted isolation of arsenical metabolites from the brown alga *Sargassum lacerifolium* (Order: Fucales; Family: Sargassaceae) afforded two new ribosides methyl 5-deoxy-5-(dimethylarsinoyl)-β-D-riboside (**344**) and 1-O-[5′-deoxy-5′-(dimethylarsinoyl)-β-D-ribosyl] mannitol (**345**), in addition to five known arsenic-containing ribosides [130].

Two batches of *Tridacna maxima* were re-analysed for additional arsenical metabolites, the first batch, collected from Exmouth in 1981, afforded three novel compounds, N-(5′-deoxy-5′-dimethylarsinoyl-β-D-ribosyloxycarbonyl) glycine (**346**), (2*S*)-3-(5′-deoxy-5′-dimethylarsinoyl-β-D-ribosyloxy)-2-hydroxypropanoic acid (**347**) and (2*R*)-3-(5′-deoxy-5′-dimethylarsinoyl-β-D-ribosyloxy)-2-hydroxypropanoic acid (**348**), as well as four dimethylarsinoylribosides reported previously (Figure 21) [131]. The second batch, collected from Exmouth in 1988, afforded two novel compounds: the arsenic-containing nucleoside 9-(5′-deoxy-5′-dimethylarsinoyl)-9*H*-adenosine (**349**), in addition to the taurine-conjugated N-4-dimethylarsinoyl)butanoyl]taurine (**350**). The second collection also afforded the previously reported (2*S*)-3-[(5′-deoxy-5′-trimethylarsonio-β-D-ribosyloxy)-2-hydroxypropyl] sulfate (**351**). The structures of **346** to **351** were validated by total synthesis [131]. The authors propose a biogenetic scheme for the formation of compounds **346** to **351** deriving from donation of all three alkyl groups present on S-adenosyl methionine to inorganic arsenate present in seawater [130].

## 3. Conclusions

Here we have reviewed the marine natural products that have been reported from the fauna and flora of Western Australian waters. This review describes the identification of over 350 metabolites representing a diverse array of chemical compounds that have been reported over the past 40 years. Most of the compounds have also been reported to display some biological activity in line with the high rates of bioactivity studies of marine natural products reported elsewhere [132,133].

**Figure 22:**
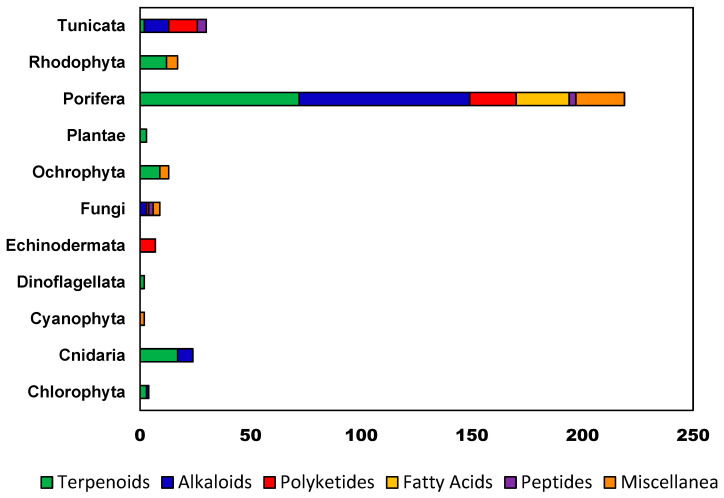
Number of metabolites isolated from Western Australian marine species, classified by taxonomy and putative biogenesis.

A statistical analysis of the distribution of marine natural products from various taxonomic sources and the percentage of each structural class among all the marine natural products reported from Western Australia (Figure 22) reveals that studies on Porifera have by far yielded the largest and most diverse number of compounds with 220 metabolites reported, from all six arbitrary biogenetic groupings. In general terms, it is likely that the quantity and relative percentage of compound classes isolated are, in part, a reflection of the biosynthetic potential of the organisms (and associated microbiota) under investigation and partly attributable to the interests of the lead researchers involved, as well as the chromatographic and analytical technology available at the time. The former observation is likely true of work conducted on Echinoderms, where all the metabolites reported from Western Australian species are polyketidic anthraquinones, a biogenic grouping known to be of chemotaxonomic relevance to the phylum [134]. The latter observation is afforded some support when analysing the extensive proportion of terpenoids isolated from Rhodophytes and Ochrophytes, work that was overwhelmingly conducted in the early 1980s by the Ghisalberti group, using predominately normal-phase column chromatography, compatible with the typically lipophilic metabolites reported. Subsequent research on alternative taxa, conducted from the 1990s to present, shows a trend towards compounds of increasingly varied biosynthetic provenance, including a higher proportion of alkaloids and polyketides, evident when analysing distributions of isolated metabolites from Porifera, Tunicata and Fungi. This trend can be explained when considering the proliferation of high- and ultra-high-performance liquid chromatography instruments, in analytical and preparative modes, as well as the propensity of researchers to operate under typically reversed-phase conditions, facilitating the purification and analysis of increasingly polar metabolites.

While there has been extensive research into marine natural products originating from other major marine biodiversity hotspots, such as the Americas, Southeast Asia, Japan, Eastern and Southern Australia and New Zealand, there have been relatively few major studies of marine natural products from Western Australia. This is the largest coastline of Australia, and biodiversity studies suggest that Western Australia marine areas are a source of significant biodiversity with many of the species remaining largely uncharacterised and underexplored [6,135,136,137]. In recent times, there have been a number of biodiversity expeditions to explore the species richness of the coastline, but chemical studies of these have so far been lacking. These recent expeditions have subsequently led to the advent of the Western Australian Marine Science Library (WAMBL), where collected specimens have been deposited for future genetic, biological and chemical analysis. The WAMBL provides researchers with easier access to specimens that were previously difficult to obtain, such as deep-sea marine sponges (>100 m). The variety of unclassified species within the WAMBL makes it of high interest for chemical and biological studies such as those we have started recently [34,53,67,75].

In an age of superbugs and viral pandemics, the need for discovering new anti-infective agents is paramount [138,139], and marine natural products are well known as a significant source of biologically active compounds [140]. To that end, the relatively underexplored chemical diversity of species occurring along the Western Australian coastline may offer many more opportunities in this area.

## Data Availability

Not applicable.
